# Exploring the Common Mechanisms of Motion-Based Visual Prediction

**DOI:** 10.3389/fpsyg.2022.827029

**Published:** 2022-03-22

**Authors:** Dan Hu, Matias Ison, Alan Johnston

**Affiliations:** School of Psychology, University of Nottingham, Nottingham, United Kingdom

**Keywords:** motion, visual prediction, spatial illusion, alpha activity, motion adaptation, individual difference

## Abstract

Human vision supports prediction for moving stimuli. Here we take an individual differences approach to investigate whether there could be a common processing rate for motion-based visual prediction across diverse motion phenomena. Motion Induced Spatial Conflict (MISC) refers to an incongruity arising from two edges of a combined stimulus, moving rigidly, but with different apparent speeds. This discrepancy induces an illusory jitter that has been attributed to conflict within a motion prediction mechanism. Its apparent frequency has been shown to correlate with the frequency of alpha oscillations in the brain. We asked what other psychophysical measures might correlate positively with MISC frequency. We measured the correlation between MISC jitter frequency and another three measures that might be linked to motion-based spatial prediction. We demonstrate that the illusory jitter frequency in MISC correlates significantly with the accrual rate of the Motion Induced Position Shift (MIPS) effect - the well-established observation that a carrier movement in a static envelope of a Gabor target leads to an apparent position shift of the envelope in the direction of motion. We did not observe significant correlations with the other two measures – the Adaptation Induced Spatial Shift accrual rate (AISS) and the Smooth Motion Threshold (SMT). These results suggest a shared perceptual rate between MISC and MIPS, implying a common periodic mechanism for motion-based visual prediction.

## Introduction

In this study we focus on the temporal properties of motion-based prediction. Prediction is a common ingredient of visual experience. For example, when you hear familiar footsteps outside the door, you will get an image of the person before you see them (Enns and Lleras, 2008). Motion-based visual prediction is more specific, referring to the ability of the visual system to predict spatial and temporal properties of moving stimuli (e.g., where and when a moving ball will be). This facility is essential to our capacity to interact with the fast-changing outside world and compensate for neural delays in visual motion processing, and programming upcoming actions (Nijhawan, 2002; Hubbard, 2005; see Hogendoorn, 2020, 2021 for recent reviews).

**FIGURE 1:**
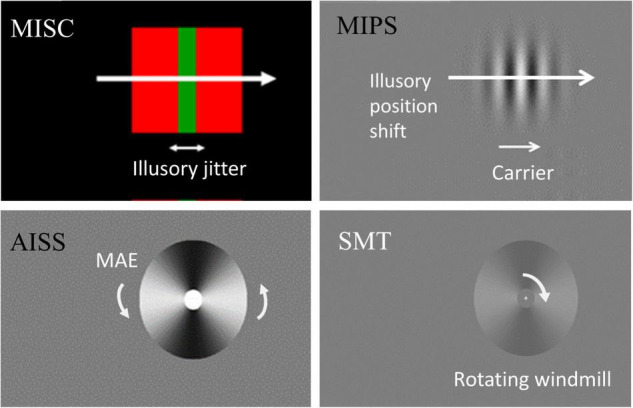
Stimulus illustration and measurement. In MISC, the equiluminant chromatic (red and green) edge and high luminance contrast (red and black) edge move together, producing an illusory jitter whose frequency is related to the MEG alpha oscillation frequency. In MIPS, the carrier movement in a static envelope leads to an illusory position shift of the envelope in the carrier motion direction. In AISS, motion adaptation induces an illusory spatial shift in the direction of the motion after-effect (MAE). In SMT, there is an upper threshold for perceiving “smooth rotation”, above which motion becomes turbulent. We measure a rate in each task – the illusory jitter frequency in MISC, the accrual rate of the illusion in MIPS and AISS, and the upper smooth motion threshold (temporal frequency) in SMT. See Supplementary Movies 1, 2 for a demonstration of the MISC and AISS illusions.

Motion-based prediction has been studied using a diverse set of tasks. Motion Induced Spatial Conflict refers to a striking effect in which an illusory jitter appears at a characteristic frequency, typically around 10 Hz, in a smoothly moving stimulus. Equiluminant and low contrast motion appears perceptually slower than high contrast luminance-based motion (Thompson, 1982; Cavanagh et al., 1984). When two edges with different apparent speeds (e.g., one perceptually slowed equiluminant chromatic or low luminance-contrast edge, one high luminance-contrast edge) move together as a combined stimulus, the central region appears to jitter (Arnold and Johnston, 2003). The jitter frequency was found to be invariant with respect to the stimulus physical speed, form, and type of motion, and perceived jitter remains when the two moving edges are presented dichoptically (Arnold and Johnston, 2003, 2005). Based on these observations, Arnold and Johnston attributed the illusion to a spatial conflict caused by visual prediction. Note that jitter disappears when there is a luminance as well as a chromatic difference at the red-green border (see Figure 1), precluding the idea that jitter arises from a non-specific modulation of the stimulus rather than intrinsic stimulus processing, and that the differences in speed have to be converted into differences in spatial position to explain the phenomenon. If the two edges in the stimulus are predicted forward to different degrees due to their differing perceived speeds, then there will be an increasing discrepancy between the spatial prediction and the upcoming stimulus, and this conflict is thought to be resolved periodically in favour of the incoming data.

The key observation in MISC is that a semi-stochastic periodic perceptual experience arises from a stimulus in constant-velocity physically-smooth motion, implying that the periodic nature of the experience arises as consequence of a recurrent neural process. Arnold and Johnston (2003) attributed this periodicity to a process of motion-based prediction, inducing a forward shift in spatial pattern, and a comparison between prediction and incoming spatial information, with a resolution of the error in favour of incoming sensory evidence over prediction. Typically, the smooth motion of luminance-based pattern would give rise to accurate predictions and consonance between prediction and incoming data with a negligible error, apart from perhaps at high speeds when, due to time pressure, there may be errors in motion computation, motion-based spatial shifts, or both. Thus, in the typical case, prediction and data will match and the recurrent process will be silent and inaccessible to experience. The proposed functional role for this processing loop is the effective calibration of velocity calculation by the visual system. However, this proposal leaves open the exact timing of the proposed predict and compare loop. The timing of the loop should be linked to the frequency of perceived jitter, but it has proved difficult to find a simple method to manipulate the frequency of the apparent jitter, which might be expected if the jitter rate is a consequence of an intrinsic property of neural processing. Though difficult to manipulate within individuals, MISC rate does vary across individuals (Minami and Amano, 2017), suggesting we may be able to explore the effect of variable processing rate using an individual differences approach.

Much of the research on visual perception has tended to treat individual differences as noise rather than a significant research tool for investigating visual processes (Peterzell and Kennedy, 2016; Mollon et al., 2017). Coren and Porac (1987) reported performance correlations among multiple spatial ability tests and the strength of visual illusions, and found that different levels of spatial abilities, as non-causal mechanisms, can predict the perceived magnitude of the illusions. More recently, Grzeczkowski et al. (2017) investigated correlations between a number of visual illusions but only found a significant correlation between the Ebbinghaus illusion and the Ponzo illusion (*R* = 0.23), both of which involve size scaling. These studies with static visual stimuli suggest a limited correlation between performance on perceptual tasks unless the phenomena are similar (e.g., size scaling). The individual differences approach has also been applied to motion processing, providing evidence that presaccadic pursuit acceleration is correlated with low-level speed discrimination whereas postsaccadic pursuit accuracy is correlated with feature tracking accuracy (Wilmer and Nakayama, 2007); motion segregation performance is predicted by the amount of spatial suppression for large stimuli (Tadin et al., 2019); spatial suppression of motion direction discrimination is also correlated with IQ (Melnick et al., 2013) and the correlation structure of duration thresholds for motion direction discrimination, as a function of spatial frequency, clusters into three spatial frequency bands (Luna and Serrano-Pedraza, 2020). If participants show similar performance across different tasks, then this implies a common element. If there is a general underlying characteristic determining performance in tasks used to study the effect of motion on perceived position, using an individual differences approach may shed light on these common elements.

It is important here to avoid comparing tasks that may lead to trivial correlations essentially by observers reconfiguring the tasks (Mollon et al., 2017). For example, vernier acuity might be reconfigured as an orientation discrimination task or a dot numerosity discrimination task may be reconfigured as a dot density discrimination task. In this case the presence of significant correlations across individuals trivially highlights the similarly of the tasks, rather than providing insights into shared component mechanisms. A strategy of comparing diverse tasks delivers the potential added benefit of linking disparate paradigms used to investigate the same basic perceptual operations.

Motion Induced Spatial Conflict is associated with a corresponding neural oscillation. Illusory jitter enhances 10 Hz oscillations in the MEG signal over that generated by physical jitter (Amano et al., 2008) and the MISC frequency is correlated with alpha frequency across individuals (Minami and Amano, 2017). An increasing body of work has demonstrated a link between perception and the frequency and phase of ongoing oscillatory activity in the alpha (8–13 Hz) and theta bands (4–8 Hz) (VanRullen and Dubois, 2011; VanRullen, 2016). Detection of near threshold stimulation modulates with the phase of alpha at the time of stimulation (Busch et al., 2009), as does the magnitude of the flash-lag effect (Chakravarthi and VanRullen, 2012) and the tendency to see a pair of flashes as double or single (Valera et al., 1981; Milton and Pleydell-Pearce, 2016). The alpha rhythm has been shown to exhibit rather large inter-subject variability (Haegens et al., 2014). Alpha frequency across individuals is correlated with, in addition to MISC frequency, the capacity to resolve two flashes (Samaha and Postle, 2015) and the rate of perceptual alternations in binocular rivalry (Katyal et al., 2019). This suggests there may be a general rate of neural processing, linked to alpha oscillation frequency, that differs across individuals.

We analyzed performance correlations between the Motion-Induced Spatial Conflict (MISC) flicker rate and rates in three other tasks – the Motion Induced Position Shift (MIPS), the Adaptation Induced Spatial Shift (AISS) and the Smooth Motion Threshold (SMT). MIPS describes the well-established observation that grating (carrier) movement in a static envelope leads to an apparent position shift of the envelope in the direction of motion (De Valois and De Valois, 1991; Arnold et al., 2007; Chung et al., 2007). Arnold and Johnston (2003) proposed that MISC arises from motion-induced shifts in spatial position (MIPS). Motion-based shifts are dynamic and take time to establish. MIPS increases rapidly over the first roughly 80 ms of stimulus presentation with a well-defined rate and then plateaus or reduces to some degree (Arnold et al., 2007; Chung et al., 2007; Jeon et al., 2020). It is therefore natural to ask if the time to establish a spatial shift (MIPS) and the time interval at which the shift and prediction diverge significantly, thought to determine MISC frequency, are related.

Adaptation Induced Spatial Shift accrual rate describes a similar effect in which motion adaptation induces an illusory spatial shift in the direction of the motion after-effect (Snowden, 1998; Nishida and Johnston, 1999; Whitaker et al., 1999; McGraw et al., 2002). After adaptation to a rotating windmill display, a static pattern appears to rotate in the opposite direction, as in the classic motion aftereffect, however, in addition to the impression of motion there is a measurable progression in the apparent location of spatial contours. The spatial shift and apparent speed of the motion aftereffect are not commensurate (Nishida and Johnston, 1999). MIPS and AISS have in common the fact that a constant motion signal in the case of MIPS, or a consistent but decreasing apparent speed in the case of the motion aftereffect, leads to an increasing spatial shift over time. This gradual increase in the size of the position shift with time implies processes of temporal integration of spatial increments. However, the motion adaptation induced effect is internally generated and much slower, with a peak displacement at around 2 s and a subsequent slow decay, compared to the around 80 ms rise in MIPS. Nevertheless, participants who show a relatively fast incremental process in AISS might also show a relatively fast incremental process in MIPS, regardless of the differences in the magnitude of the motion signal. We measured the accrual rate of the illusion (magnitude change over time) in both tasks. If MIPS and AISS involve a unitary process of motion-based visual prediction, which is also shared by MISC, we might expect to see rate correlations among these tasks.

Smooth Motion Threshold is an upper speed threshold after which smooth motion gives way to an apparent turbulent flickering motion (Harris, 1984). If it is the case that smooth motion requires accurate visual prediction and matching of prediction and incoming spatial pattern, with turbulent motion resulting from an inability of prediction to track the spatial pattern of the moving stimulus at high speed, we may expect the SMT to also reflect some general rate of motion prediction and comparison.

Our results showed a significant positive correlation between MISC frequency and MIPS accrual rate, but there was no significant correlation between the rate measures for any other pair of tasks. The findings offer an intriguing possibility of the existence of a common and periodic mechanism in motion-based visual prediction.

## Materials and Methods

### Participants

Thirty-one subjects with normal or corrected-to-normal visual acuity participated in the study. They were two of the authors, eight staff and Ph.D. students in vision science, and 21 additional participants who were naïve to psychophysics. Three naïve subjects, amongst those participating, were excluded from the analyses. One participant reported that they could not perceive the illusory jitter in MISC, another participant always made the same response in the MISC task, although self-reported that they could see the jitter, and a third participant withdrew and did not complete the AISS task. Therefore, a total of 28 participants (aged 16–64 years, mean age 28.64; std 8.75, 13 male) were included. The study was approved by the Ethics Committee in School of Psychology, University of Nottingham. All the participants gave written informed consent or guardian consent before taking part.

### Apparatus

All stimuli were generated using Bits# (Cambridge Research System, Cambridge, United Kingdom) and presented on 20-inch CRT monitor (Mitsubishi DPlus 230SB) with 1024 x 768 resolution and 120 Hz refresh rate. Bits++ Mode was used, and Gamma correction was achieved using a Colorcal (Cambridge Research System, Cambridge, United Kingdom). The stimuli were viewed at a distance of 114 cm, with the participant head stabilized by a head and chin rest. The computer operating system was Ubuntu 18.04. The programming platform was Psychotoolbox-3 (PTB-3; Kleiner et al., 2007) on MATLAB (The Mathworks, Natick, MA, United States; Version Matlab_R2018a). The experimental room was completely dark, except for the stimuli.

### General Methods

Data collection for the four tasks (MISC, MIPS, AISS and SMT) took about 3–4 h, which was completed in 3–4 separate days within two consecutive weeks. On one test day, a standard test order was MISC first and AISS last, with MIPS and SMT randomly arranged. This procedure was used to avoid the effects of dark adaptation on MISC, and the influence of fatigue caused by the AISS tests on other tasks (i.e., each test run of AISS took about 30 min while that of the other tasks took about 10 min; see test details below for each task). On some later test days, once data collection for MISC and SMT has been completed, we tested MIPS first then AISS. Participants practiced before the formal test for each task (usually 5–10 trials in MISC, AISS and SMT, and one run of MIPS). Throughout all the tests, participants were required to fixate on the centre cross while viewing the stimuli.

We include stimulus illustrations in Figure 1, and a summary of stimulus and task parameters in Table 1.

**TABLE 1:** Stimulus and task parameters for the four tasks in this study.

	MISC	MIPS	AISS	SMT
Stimulus type	Red squares with superimposed green or black bar	Gabor patches with drifting gratings (carriers)	Windmill(s) created by a sinusoidal luminance modulation of 2	
			cycles per rotation	
Stimulus geometry (deg)	Red square: 2.9 × 2.9 Green/black bar: 0.5 × 2.9 Vertical eccentricity: 3.6 Horizontal eccentricity: 0–7.0 in motion Fixation cross: 0.3 × 0.3 Black background centred size: 17.0 × 12.8	Grating size: 1.44 Grating spatial frequency: 2.0 c/deg Grating temporal frequency: 4.68 c/s Gabor mask sigma: 0.23 Grating horizontal eccentricity: 4.0 Comparison grating vertical eccentricity:1.5 Fixation cross: 0.3 × 0.3	Windmill diameter: 4.0 Windmill central hole diameter: 0.67 Windmills eccentricity: 4.0 Fixation cross: 0.3 × 0.3	Windmill diameter: 8.0 Windmill central hole diameter: 1.33 Fixation cross: 0.3 × 0.3
Stimulus luminance (cd/*m*^2^)	Red square: 23.2 Green bar: 20.7–35.4 Black background: 0 White edges: 98.5	Gratings: 9.9–88.6 Background: 49.3	Windmill: 9.9–88.6 Background: 49.3	Windmill: 39.4–59.1 Background: 49.3
Stimulus contrast	∼1	0.8	0.8	0.2
Motion parameters	Linear movement speed: 7.0 deg/s Black bar physical jitter: sinusoidal; amplitude – 0.1 deg; frequency – see “Manipulation”	Drift speed: 2.34 deg/s Test drift direction: leftward Comparison drift direction: rightward	3.0 rotation/s (6.0 Hz)	See “Manipulation”
Stimulus duration (s)	2.0	See “Manipulation”	Adaptation phase: 180 Top-up: 8.0 The test: 2.2 The comparison: 0.2	2.0
Method	Constant stimuli and 2AFC	Staircase (1-up 1-down or 1-down 1-up)		Constant stimuli and 2AFC
Manipulation	Physical jitter frequency: 5.0, 7.1, 8.0, 8.6, 9.2, 10.0, 10.9, 12.0, 13.3, 15.0 Hz	Stimulus duration: 16.7–183.3 ms in 16.7 ms increments; also, 500 and 1000 ms, resulting in 12 staircases. To measure the magnitude of the illusion in each staircase, the physical position of the test changed, while the comparison positions kept invariant.	T-C interval (the illusion accumulation time): 0–2 s in 0.5 s increments, resulting in 5 staircases. To measure the magnitude of the illusion in each staircase, the test orientation changed, while the comparison was always vertical.	Windmill temporal frequency (defined by luminance change; 1 cycle/s of windmill rotation delivers a pixel temporal frequency of 2 Hz): 4.0, 5.3, 6.0, 6.7, 7.3, 8.0, 9.3, 10.0 Hz
		In a single test run, all the staircases were intermingled,		
		and each randomly started from a very high or low value.		
Trials per test run	10 for each frequency condition	10–20 per staircase	10–15 per staircase	10 for each temporal frequency condition
Test runs	1–5	At least 3	At least 3	1

*Michelson contrast was specified throughout; 2AFC = two-alternative forced choice; also see Figure 1.*

### Motion Induced Spatial Conflict

The stimuli parameters were the same as used by Minami and Amano (2017). For the illusory jitter stimulus (see Figure 1), a vertical green bar [0.5 × 2.9 deg (W × H)] was superimposed on a red square [2.9 × 2.9 deg (W × H)]. The red colour was always at the maximum luminance the monitor could generate, while the green colour (0.42 ± 0.05; range: 0.31–0.54 on 0–1 RGB scale) was adjusted for each participant before the formal experiment to make this perceptually equiluminant with the red, using a minimum motion task (Cavanagh et al., 1987; see task details in Supplementary Material 1). The green bar and red square, as a combined stimulus, was positioned vertically 3.6 deg away from the centre cross, and moved together either from the left or right end of the screen (starting horizontally 7 deg away from the centre cross), at a speed of 7 deg/s. Participants were required to maintain fixation, as tracking the stimulus eliminates the illusion (see Supplementary Movie 1 for a demonstration of the illusion). The stimulus with physical jitter was the same as the green/red stimulus, except that the green bar was replaced by a same-sized black bar jittering physically according to a sin function (position changed over time; amplitude fixed at 0.1 deg). The background was black (centred size: 17.0 × 12.8 deg) except for small white surround, which served to maintain an approximately constant level of dark adaptation through the task.

To measure the illusory jitter frequency in MISC, a constant stimuli binary choice task was used. After presenting both illusory and physical jitter stimuli simultaneously for 2 s (the stimuli moved laterally in opposite directions, one above and one below fixation), participants reported “which one jitters faster” by pressing the “UP” or “DOWN” button on the computer keyboard. The physical jitter frequency randomly ranged across 10 levels (5.0, 7.1, 8.0, 8.6, 9.2, 10.0, 10.9, 12.0, 13.3, 15.0 Hz), and each frequency level was repeated 10 times, resulting in 100 trials in total for a test run. The relative position of the illusory jitter to physical jitter (one above and one below fixation) was randomly changed across trials. In addition, there was a 2 s interval between each trial, during which the centre cross remained on the screen. The duration of each test run was about 10 min. Each participant received 1–5 test runs. We increased the number of runs for naïve participants in order to improve the precision of the measurement, given the likelihood of greater variability in naïve participants. The average number of test runs for naïve participants and expert psychophysical observers were 2.8 and 1.9, respectively.

### Motion Induced Position Shift

The stimuli consisted of three vertically presented Gabor patches (width/height, 1.44 deg; spatial frequency, 2.0 c/deg; temporal frequency, 4.68 c/s; sigma in x and y, 0.23 deg; peak contrast 80%), with static envelopes and moving carriers, presented on a grey background (see Figure 1 for one stimulus). The top and bottom patches (the comparisons) were always vertically aligned and placed 4 deg horizontally away from the centre cross. The vertical eccentricity for the comparisons was 1.5 deg, and for the middle test patch, 0 deg. The three patches moved at the same speed of 2.34 (4.68/2) deg/s, but in opposite directions - the comparisons always moved to the right while the middle test carrier always moved to the left.

A staircase procedure was used to measure the magnitude of the illusion for different stimuli durations. The test duration varied across 12 levels (16.7, 33.3, 50.0, 66.7, 83.3, 100.0, 116.7, 133.3, 150.0, 183.3, 500, 1000 ms), resulting in 12 staircases. The three Gabors were randomly presented either on the left or right side of fixation a) to control for any bias related to field of view, (b) to reduce potential drifts in fixation and (c) to balance effects of movement direction (toward or away from the fovea). The three Gabors had the same duration within a trial, and the test duration was randomized over trials. After each presentation, participants reported if the position of the middle test patch was more to the left or right of the upper and lower patches, by pressing the “LEFT” or “RIGHT” button on the computer keyboard. For each staircase, the physical position of the test patch was changed by a simple up-down method (Levitt, 1971): if the participant’s last response were “LEFT”, the test position would be more right relative to the position in the last trial; if the last response were “RIGHT”, the test position would be more left. A reversal occurred when the participant’s last two responses were different (e.g., pressed “LEFT” followed by “RIGHT”). The increment of the test position (the step size) halved with an increase in reversal number, starting from 0.16 deg when the reversal number was zero, and saturating at 0.04 deg after the reversal number reached two. There were six reversals in total for each staircase. All the 12 staircases were intermingled in one experimental run to reduce potential response bias caused by participants’ recognition of a single test staircase, with each staircase (a) starting from a very high or low value chosen at random and, (b) waiting at the nth reversal point until all the other staircases reached that point. Each participant completed at least three experiment runs.

### Adaptation Induced Spatial Shift

The stimuli and methods were the same as in Nishida and Johnston (1999). Windmills with a 40 min grey region at the centre subtended 4 deg in diameter and were presented 4 deg left or right of a central fixation cross. Each windmill had a sinusoidal luminance modulation of 2 cycles per rotation at 80% contrast on a grey background (see Figure 1 and Supplementary Movie 2).

During an initial adaptation phase, participants watched a windmill rotating at 3 rotations/s (6 Hz) on the left side of the screen for 3 min (the stimulus was the same as described above). In each trial of the following test phase, a static test windmill was presented for 2.2 s on the left at the same position as the adapter, and a comparison windmill was presented for 0.2 s on the opposite side (4 deg right to the centre) simultaneously, both with a sharp onset and offset. The onset time between the test and comparison (T-C interval) determined the duration of the spatial shift at the test position which varied across five levels (0, 0.5, 1.0, 1.5, 2.0 s). Participants indicated, at the time that the comparison was presented, if the black region of the left test windmill was more clockwise or anti-clockwise relative to that of the right comparison windmill (which was always vertical) by pressing the “LEFT” or “RIGHT” button on the keyboard. The use of a relative judgment allowed us to discount any influence of eye movements. Except for the first 3-min adaptation period, there was always 8 s top-up adaptation period preceding each new test trial.

A similar staircase procedure as in MIPS was used to measure the magnitude of the spatial shift on each T-C interval condition. For each condition (or staircase), the physical orientation of the test windmill was changed according to participant’s last response in a simple up-down fashion: a response of “the test more clockwise relative to the comparison stimulus” led to the test orientation more “anti-clockwise” in the next trial, and vice versa. A reversal occurred when the responses in two consecutive trials were different. The step size halved with an increase in reversal number, starting from eight angular degrees when the reversal number was zero, and saturating at one angular degree after the reversal number reached three. Six reversals in total were measured for each staircase. To equate the state of adaptation, the staircases were intermingled, with the same setting as in MIPS. Each participant completed at least three runs.

### Smooth Motion Threshold

The same windmill as was used for AISS was used here, except that the size of the windmill was doubled, and the contrast reduced to 20% (see Figure 1).

The method of constants was used to measure the smooth motion threshold. The windmill was alternately rotated in the clockwise or anti-clockwise direction on different trials, at one of a set of randomly chosen temporal frequencies (ranging across 8 levels: 4.0, 5.3, 6.0, 6.7, 7.3, 8.0, 9.3, 10.0 Hz). Note that 1 cycle/s of windmill rotation delivers a pixel temporal frequency of 2 Hz. There were 5 repetitions for each frequency condition and two directions of rotation, leading to 80 (8 × 5 × 2) trials in total. In each trial, the rotating windmill was presented for 2 s then disappeared. Participants judged if the presented stimulus was moving smoothly or not, by pressing the “J” or “K” button on a standard keyboard. A lack of smooth motion appeared as flicker during the motion sequence. This distinction was explained in the instructions provided to participants before the experiment started.

## Results

### Results in Each Task

**FIGURE 2:**
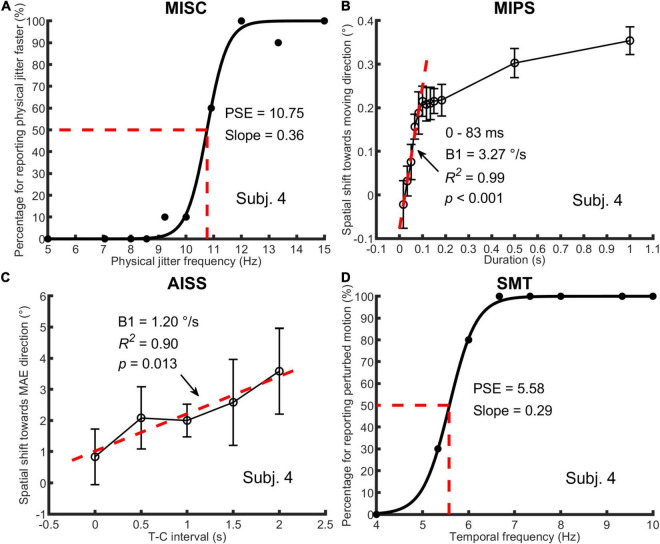
An illustration of one participant’s data in all four tasks. In **(A)** MISC, the percentage of trials for “perceiving physical jitter faster” is plotted as a function of physical jitter frequency. Logistic functions [*y* = 1/(1+exp(*a*-x)/*b*)] were fitted to the data, using a least squares method. The PSE and slope, which are *a* and *b* in the function, represent the illusory jitter frequency and participant discrimination performance, respectively. In **(B)** MIPS and **(C)** AISS, the magnitude of the illusion (mean and standard deviation based on the data of all test runs) is plotted as a function of stimulus duration or T-C interval. A linear model (*y* = B0 + B1 × t) was used to fit the first five mean values (0–83 ms) in MIPS and all the mean values in AISS. The B1 stands for the accrual rate of the illusion in the model. The R-squared and *p*-value in the regression analysis are shown in the figure. Note that the unit of the illusion (°) is retinal degrees in MIPS and angular degrees in AISS, respectively. In **(D)** SMT, the analysis method was the same as for MISC, except that the percentage of trials for “perceiving perturbed motion” is plotted as a function of stimulus temporal frequency, and the PSE indicates the smooth motion threshold. Also see Figure 1 and Table 1.

The data for one representative participant in each of the four tasks with model details are plotted in Figure 2. Descriptive statistics for all measures are listed in Table 2 (see also Supplementary Material 2 for all participant results). We describe how the data was treated for each task below.

**TABLE 2:** Descriptive statistics for all measures.

Task	Measure	Mean	Median	SD	Range	R^2^ range	*n*
MISC	Illusory jitter frequency (Hz)	9.36	9.12	0.92	8.15–10.75		28
MIPS	Accrual rate – selected observers (retinal deg/s)	2.80	2.70	0.84	1.23–5.37	0.75–0.99	26
	Accrual rate – all (retinal deg/s)	2.67	2.65	0.93	0.83–5.37	0.46–0.99	28
AISS	Accrual rate – selected observers (angular deg/s)	1.48	1.16	0.72	0.57–3.46	0.79–0.97	14
	Accrual rate – all (angular deg/s)	1.01	1.12	0.84	−0.74–3.46	0.08–0.97	28
SMT	Temporal frequency (Hz)	6.46	6.66	0.52	5.54–7.40		28

*As a linear regression model was used to calculate the accrual rate in MIPS and AISS, results are reported including all participants (all) and those where the regression was significant (selected observers, significance level: α = 0.05). The ranges of R-squared in the regression are given in the table.*

In MISC and SMT, logistic functions were fitted to participant response data in each test run. The point of subjective equality (PSE) is defined as the 50% point in the function, representing the illusory jitter frequency in MISC and smooth motion threshold in SMT, respectively. For participants who completed more than one test run, their combined result was the average of the results across runs.

In MIPS and AISS, only the last 4 (out of 6) reversals in a single staircase were processed, and all staircase results in different test runs were put together to calculate the magnitude of the illusion (the mean of all results) on each manipulated time condition. Linear models were fitted to the mean values in Matlab using the “regress” function (Chatterjee and Hadi, 1986). Only the first five mean values (0–83 ms) in MIPS were used since all participant data indicated a saturation of the illusion at about 100 ms (see Supplementary Material 3). All the mean values were used in AISS since there was no saturation (see Supplementary Material 4). The regression coefficient in the model represents the accrual rate of the illusion.

### Correlations

**FIGURE 3:**
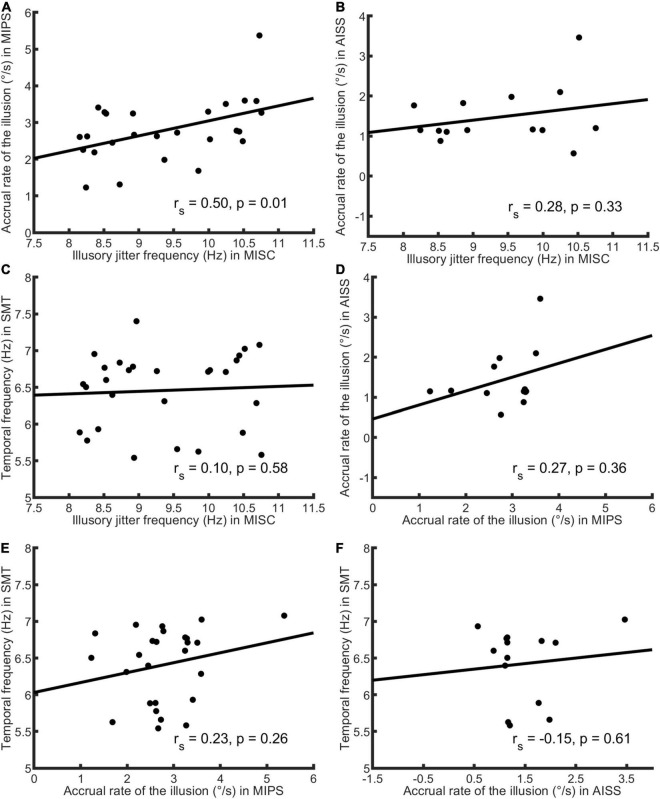
Scatter plots of participant performance data for all pairings of the four tasks. Spearman’s *r* and *p*-value calculated by each paired data are shown in each figure. Only significant accrual rates in MIPS and AISS were used (see Figure 2 for details), and the sample size was 28 in the MISC and SMT task, 26 in MIPS, and 14 in AISS. **(A)** The plot of MISC and MIPS scores. The illusory jitter frequency in MISC and accrual rate in MIPS were significantly correlated (*N* = 26). There seems to be an outlier for the MIPS data (5.37°/s, on the figure’s top-right corner). Although, Spearman’s correlation is rank-based and has an advantage of robustness to outliers, we also conducted the correlation analysis while excluding this outlier. The result was still significant (Spearman’s *r* = 0.44, *p* = 0.03, *N* = 25). **(B)** The plot of MISC and AISS scores. **(C)** The plot of MISC and SMT scores. **(D)** The plot of MIPS and AISS scores. **(E)** The plot of MIPS and SMT scores. **(F)** The plot of AISS and SMT scores. All correlations in **(B–F)** were non-significant. Note the lines through the data were fitted using linear regression, whereas Spearman’s correlation is based on rank order, and therefore the slopes and the signs of the *r*-values need not correspond.

First, we analysed the data using only the significant accrual rates (i.e., *p* < = 0.05 in the regression) in MIPS and AISS. Given the small sample sizes (the lowest: *N* = 14 in AISS), the Shapiro-Wilk (S-W) test was used to check the data normality in each task. The result showed that none of the performance data follows the normal distribution (all *p* < 0.05). Hence, Spearman’s correlation was employed for the correlation analysis. Spearman’s correlation is in any case to be preferred, since it is based on rank and is therefore is more robust to outliers (Mollon et al., 2017). Table 3 shows the results: MISC and MIPS scores were significantly correlated (Spearman’s *r* = 0.50, *p* = 0.01; 95% Bootstrap CI = (0.12, 0.75), see details in section “Result reliability test”); all other correlations were non-significant (all *p* > 0.05). Since there seems to be an outlier in the MIPS data (5.37 deg/s), we further analysed the MISC and MIPS correlation with this outlier excluded. A significant correlation between the two task performances remained (Spearman’s *r* = 0.44, *p* = 0.03, *N* = 25). Figure 3 shows scatter plots of the participant performance data for all pairs of the four tasks.

**TABLE 3:** Correlations using significant accrual rates only in MIPS and AISS.

Task	MISC	MIPS	AISS	SMT
MISC	—			
MIPS	0.50**	—		
AISS	0.28	0.27	—	
SMT	0.10	0.23	−0.15	—

Spearman’s correlation was used; the sample size was 28 in MISC and SMT, 26 in MIPS, and 14 in AISS, respectively; ** p = 0.01; see also Figure 3.

As only five mean values were used to build the linear model in MIPS and AISS, the requirement that the accrual rate reach significance might be considered too strict. We further analysed the data including all the 28 participants’ results for each task. The S-W test showed that the performance data in most tasks did not follow the normal distribution (all *p* < = 0.05 except *p* = 0.11 for AISS). We again used Spearman correlation. Table 4 shows the results: only the performance in MISC and MIPS were significantly correlated (Spearman’s *r* = 0.47, *p* = 0.01); all other correlations were non-significant (all *p* > 0.05). We also calculated the correlation between the MISC and MIPS performance while excluding the apparent outlier in MIPS (5.37 deg/s). A significant correlation between the two task performances remained (Spearman’s *r* = 0.41, *p* = 0.04, *N* = 27). Supplementary Material 5 shows scatter plots of the participant performance data for any pair of the four tasks.

**TABLE 4:** Correlations using all accrual rates in MIPS and AISS.

Task	MISC	MIPS	AISS	SMT
MISC	—			
MIPS	0.47**	—		
AISS	0.12	0.28	—	
SMT	0.10	0.06	−0.22	—

Spearman’s correlation was used; the sample size was 28 for each task; ^**^ p = 0.01; see also Supplementary Material 5.

### Reliability Test

To test the reliability of the significant relationship between MISC and MIPS with respect to sample size (maximum *N* = 28), we used a bootstrapping method (Efron and Tibshirani, 1986). For this analysis, we included the data from 26 subjects with significant accrual rates (by the linear model) in MIPS.

**FIGURE 4:**
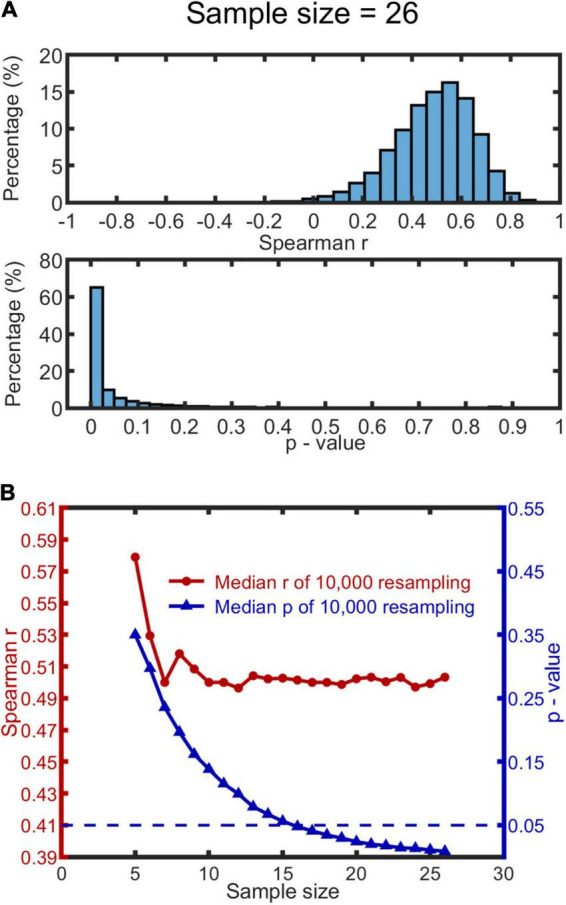
Bootstrapping results based on MISC and significant MIPS data (*N* = 26). 10,000 bootstrap resamples were drawn for each condition of sample size (range: 5–26), resulting in 10,000 Spearman’s *r* and *p*-value for each. **(A)** A representative histogram of *r* and *p*-values when the sample size is 26. The histograms look similar when the sample size ranges from 10 to 26; see all the histograms in Supplementary Material 6. **(B)** The red and blue curve show the change of the median *r* and *p*-value (based on the 10,000 resamples) as a function of sample size, respectively. The blue dashed line represents *p* = 0.05.

We used the following procedure: a sample size was first determined within a range of 5 to 26; then for each level of sample size, we drew 10,000 bootstrap samples from the participant data, and calculated Spearman’s *r* and *p*-value for each sample. Outliers existed in both *r* and *p*-values distributions (see Figure 4A for a representative histogram when the sample size is 26, or Figures in Supplementary Material 6 for all histograms). We therefore used the median, rather than mean, to measure the central tendency of the data. We report the median of the 10,000 *r* and 10,000 bootstrap *p*-values as a function of sample size (see Figure 4B). The median *r* appears to saturate when the sample size reaches 10, with the *r* at 0.50. The median *p*-value function drops below 0.05 when the sample size exceeds 15 (*p*-value of 0.056). We conclude from this that our sample size (*N* = 26) was sufficient to detect a significant correlation.

## Discussion

In this study, we adopted an individual differences approach to investigate relationships amongst four tasks (see Figure 1) within the framework of motion-based visual prediction. In the MISC task, we measured the illusory jitter frequency that has been shown to be correlated with and match the frequency of alpha oscillations in the brain (Amano et al., 2008; Minami and Amano, 2017). In the MIPS task, we measured the accrual of the positional shift in a stimulus duration range of 0–83 ms, using a linear regression model. We showed that the measures in these two tasks were significantly correlated, regardless of whether we included just the significant accrual rates (by the linear model) in MIPS (*r* = 0.50, *p* = 0.01, *N* = 26), or not (using all the data; *r* = 0.47, *p* = 0.01, *N* = 28). The robustness of the significant result against sample size was further confirmed by a bootstrapping method. In addition, a scatter plot of the naïve participant and expert psychophysical observer data on the two tasks showed that it is unlikely that the significant correlation was a spurious result arising from pooling of two non-overlapping clusters based on expertise (Makin and Orban de Xivry, 2019; see Supplementary Material 7). Overall, these results suggest a shared mechanism in MISC and MIPS, implying a common, periodic process in motion-based visual prediction.

We did not see any correlations between performance in the SMT task and the other tasks. There is no evidence for a link between the upper threshold for smooth motion and the frequency of illusory jitter. However, the lack of a correlation of SMT with other tasks indicates that the link between MISC and MIPS is not simply a consequence of non-perceptual generic factors such as personality, arousal, conscientiousness, expertise, or the ability to maintain a stable response criterion, varying across participants – if so, we might expect correlations between all tasks.

Researchers do not typically differentiate between adaptation- and real-motion-induced spatial illusions (Whitaker et al., 1999; McGraw et al., 2002; Arnold et al., 2007; Chung et al., 2007). However, our result showed that the performance in the AISS task was not correlated with that in the MIPS or MISC task. It is possible that separate mechanisms may underly these illusions arising from real and illusory motion. However, there are a number of potential alternative reasons for the lack of correlation. The first is that the measuring AISS using a temporal probe technique is more difficult for naïve participants and the data is therefore likely to be noisier than the MIPS data. Second, the speed of seen motion is substantially higher for real motion than for the motion after-effect. Thirdly, the time scales of these effects are very different (0–2 s in AISS vs. 0–83 ms in MIPS vs. instantaneously in MISC, see Figure 2 and Supplementary Materials 3, 4 for participant data in AISS and MIPS).

The observed positive correlation between MISC and MIPS timing suggests they both reflect a common rate of neural processing. However, the mechanisms by which motion influences perceived position and spatial pattern remain unclear. Two related approaches have been taken to linking representations of motion and position. Both approaches are in essence models of object tracking in which object trajectories are coded in terms of a representation of object position over time. The first strategy is motivated by the observation that a joint representation of motion and position can support a solution to the aperture problem by augmenting a probabilistic velocity distribution with position and using both to constrain a predictive model, thereby using trajectories over time to limit possible solutions (Perrinet and Masson, 2012). This approach has been extended to model position extrapolation in trajectory estimation (Khoei et al., 2013) and the flash-lag effect (Khoei et al., 2017). The second approach combines estimates of texture motion and object motion and provides a model of MIPS by changing the relative weights of texture motion and window (object) motion (Kwon et al., 2015), the latter of which would be zero in the case of static windows. Although both of these approaches allow a shift in the representation of position, only velocity and position are represented in the models; they do not address a shift in spatial pattern. The proposal that motion-based shifts involve the forward prediction of spatial pattern is supported by evidence of subthreshold summation of predictions and in-phase spatial pattern at the leading edge of motion (Arnold et al., 2007; Roach et al., 2011).

Motion Induced Position Shift increases over the first 80 ms of stimulation and is then sustained over time. After this time point the dynamic system is essentially in a stationary mode in that the stimulus velocity and the consequent spatial shift are approximately constant as indeed will be the relationship between motion-based spatial prediction and the sensory input. We note that the magnitude of the shift can reduce at longer durations and higher speeds (Chung et al., 2007; Jeon et al., 2020), however, this may reflect rapid motion adaptation, or better segmentation of the window from the moving carrier. The spatial jitter in MISC also implies a shift of spatial pattern, however, the shift changes too quickly to be directly measured. The correlation between the accrual rate in MIPS and the jitter frequency in MISC suggests that the spatial shift in the high contrast border may accrue at the same rate in both stimulus types within the first 100 ms. However, in MIPS there is a match between the motion-based prediction and the upcoming stimulus, whereas in MISC, because the equiluminant and luminance-contrast speed signals differ, it is thought that there is an increasing discrepancy in the motion-based spatial prediction over time which is resolved in favour of the incoming data before the cycle is repeated. This proposal would link the MISC cycle frequency with the accrual rate of motion-based spatial shifts.

Note that a velocity signal does not contain any information about the spatial pattern that gives rise to it and therefore its influence on spatial pattern and its apparent position needs to be on a separate, more low-level, spatial representation. Further indications of an interaction between processing levels in the motion system comes from evidence that adaptation for global motion has both global and local components (Scarfe and Johnston, 2011). To explain the link between MISC and alpha frequency we propose a plausible functional circuit between MT/V5 in which speed and direction of motion may be encoded and represented, and V1 which contains information about the spatial pattern (Nishida and Johnston, 1999; McGraw et al., 2002; Chung et al., 2007; also see a review of MT neurophysiology in Born and Bradley, 2005). MT/V5 has also been shown to be necessary for Representational Momentum – a typical result of motion-based visual prediction (Senior et al., 2002; see a review of the effect in Hubbard, 2005). The specific process in a predict-and-compare loop could be that MT/V5 sends motion signals (e.g., about velocity) to V1, then V1 generates the predicted spatial representation, compares the prediction to the instantaneous input spatial pattern at the appropriate time, and returns a prediction error (if any) to MT/V5, which is then used to calibrate motion processing (Johnston, 2013, 2017). Given that MIPS is established by 80 ms, the interactions between motion coding and spatial representation would need to operate at a high rate. We expect the checking process to be recursive and discrete as the (delayed) motion signal from MT/V5 would have to be applied to a stored spatial signal in V1 corresponding to the time the motion calculation was initiated and then compared with the incoming spatial representation. The proposed recursive interaction between two brain areas could generate both MISC and oscillating brain activity in the alpha frequency band (Amano et al., 2008; Minami and Amano, 2017; Alamia and VanRullen, 2019), or alternatively activity at the alpha frequency might control the timing of the operations. The correlation between MISC and MIPS timing offers the interesting possibility of an additional link between alpha frequency and MIPS accrual rate, which merits further investigation.

## Data Availability Statement

The raw data supporting the conclusions of this article will be made available by the authors, without undue reservation.

## Ethics Statement

The studies involving human participants were reviewed and approved by Ethics Committee, School of Psychology, University of Nottingham. Written informed consent from the participants, or from the participants legal guardian/next of kin, was provided to participate in this study.

## Author Contributions

DH and AJ designed the experiment and wrote the first draft of the manuscript. DH collected the data. All authors analysed the data, read, and revised the manuscript and approved the submitted version.

## Conflict of Interest

The authors declare that the research was conducted in the absence of any commercial or financial relationships that could be construed as a potential conflict of interest.

## Publisher’s Note

All claims expressed in this article are solely those of the authors and do not necessarily represent those of their affiliated organizations, or those of the publisher, the editors and the reviewers. Any product that may be evaluated in this article, or claim that may be made by its manufacturer, is not guaranteed or endorsed by the publisher.

## Supplementary Material

Supplementary Movie 1 This movie demonstrates the MISC illusion. When fixating on the centre cross, the illusory jitter appears around the green and red edge position, along the axis of movement, for both top and bottom stimuli. The jitter illusion should disappear when either the upper or lower stimulus is tracked. The illusion requires the green and red colour to be perceptually equiluminant for the observer and is clearest at moderately low ambient luminance levels. The relative luminance levels of the red and green segments can sometimes be adjusted by tilting the display screen. For the clearest demonstration download the video and play it on a local computer.

Supplementary Movie 2 This movie provides a demonstration of the AISS illusion. After 30 s adaptation, two static windmills are presented on the screen for 2 s – one on the left and one on the right side. The left static stimulus appears to rotate anti-clockwise, and the right appears to rotate clockwise. The illusory shift of the dark vertical bar increases gradually over around 2 s. After the first adaptation period, there is a 5 s top-up adaptation period preceding the next appearance of the two static windmills. To see the illusion, please maintain fixation on the centre cross while watching the video. We recommend downloading the video and playing it on a local computer.

## References

[B1] Alamia A. VanRullen R. (2019). Alpha oscillations and traveling waves: signatures of predictive coding? *PLoS Biol.* 17:e3000487. 10.1371/journal.pbio.3000487 31581198 PMC6776260

[B2] Amano K. Arnold D. H. Takeda T. Johnston A. (2008). Alpha band amplification during illusory jitter perception. *J. Vis.* 8:3. 10.1167/8.10.3 19146345

[B3] Arnold D. H. Johnston A. (2003). Motion-induced spatial conflict. *Nature* 425 181–184. 10.1038/nature01955 12968181

[B4] Arnold D. H. Johnston A. (2005). Motion induced spatial conflict following binocular integration. *Vis. Res.* 45 2934–2942. 10.1016/j.visres.2005.04.020 16139322

[B5] Arnold D. H. Thompson M. Johnston A. (2007). Motion and position coding. *Vis. Res.* 47 2403–2410. 10.1016/j.visres.2007.04.025 17643464

[B6] Born R. T. Bradley D. C. (2005). Structure and function of visual area MT. *Annu. Rev. Neurosci.* 28 157–189. 10.1146/annurev.neuro.26.041002.131052 16022593

[B7] Busch N. A. Dubois J. VanRullen R. (2009). The phase of ongoing EEG oscillations predicts visual perception. *J. Neurosci.* 29 7869–7876. 10.1523/JNEUROSCI.0113-09.2009 19535598 PMC6665641

[B8] Cavanagh P. MacLeod D. I. Anstis S. M. (1987). Equiluminance: spatial and temporal factors and the contribution of blue-sensitive cones. *JOSA A* 4 1428–1438. 10.1364/josaa.4.001428 3625323

[B9] Cavanagh P. Tyler C. W. Favreau O. E. (1984). Perceived velocity of moving chromatic gratings. *JOSA A* 1 893–899. 10.1364/josaa.1.000893 6470841

[B10] Chakravarthi R. VanRullen R. (2012). Conscious updating is a rhythmic process. *Proc. Natl. Acad. Sci. U.S.A.* 109 10599–10604. 10.1073/pnas.1121622109 22689974 PMC3387058

[B11] Chatterjee S. Hadi A. S. (1986). Influential observations, high leverage points, and outliers in linear regression. *Stat. Sci.* 1 379–393.

[B12] Chung S. T. Patel S. S. Bedell H. E. Yilmaz O. (2007). Spatial and temporal properties of the illusory motion-induced position shift for drifting stimuli. *Vis. Res.* 47 231–243. 10.1016/j.visres.2006.10.008 17190608 PMC2734886

[B13] Coren S. Porac C. (1987). Individual differences in visual-geometric illusions: predictions from measures of spatial cognitive abilities. *Percept. Psychophys.* 41 211–219. 10.3758/bf03208220 3575080

[B14] De Valois R. L. De Valois K. K. (1991). Vernier acuity with stationary moving Gabors. *Visi. Res.* 31 1619–1626. 10.1016/0042-6989(91)90138-u 1949630

[B15] Efron B. Tibshirani R. (1986). Bootstrap methods for standard errors, confidence intervals, and other measures of statistical accuracy. *Stat. Sci.* 1 54–75.

[B16] Enns J. T. Lleras A. (2008). What’s next? New evidence for prediction in human vision. *Trends Cogn. Sci.* 12 327–333. 10.1016/j.tics.2008.06.001 18684660

[B17] Grzeczkowski L. Clarke A. M. Francis G. Mast F. W. Herzog M. H. (2017). About individual differences in vision. *Vis. Res.* 141 282–292. 10.1016/j.visres.2016.10.006 27919676

[B18] Haegens S. Cousijn H. Wallis G. Harrison P. J. Nobre A. C. (2014). Inter-and intra-individual variability in alpha peak frequency. *Neuroimage* 92 46–55. 10.1016/j.neuroimage.2014.01.049 24508648 PMC4013551

[B19] Harris M. G. (1984). The role of pattern and flicker mechanisms in determining the spatiotemporal limits of velocity perception. 1. Upper movement thresholds. *Perception* 13 401–407. 10.1068/p130401 6527927

[B20] Hogendoorn H. (2020). Motion extrapolation in visual processing: lessons from 25 years of flash-lag debate. *J. Neurosci.* 40 5698–5705. 10.1523/JNEUROSCI.0275-20.2020 32699152 PMC7380963

[B21] Hogendoorn H. (2021). Perception in real-time: predicting the present, reconstructing the past. *Trends Cogn. Sci.* 26 128–141. 10.1016/j.tics.2021.11.003 34973925

[B22] Hubbard T. L. (2005). Representational momentum and related displacements in spatial memory: a review of the findings. *Psychon. Bull. Rev.* 12 822–851. 10.3758/bf03196775 16524000

[B23] Jeon H.-J. Yun Y. Kwon O.-S. (2020). Integration of position and predictive motion signals in aging vision. *Sci. Rep.* 10 1–11. 10.1038/s41598-020-65568-y 32472035 PMC7260223

[B24] Johnston A. (2013). “Visual time perception,” in *The New Visual Neurosciences*, eds Werner J. S. Chalupa L. M. (Cambridge, MA: MIT Press), 749–762.

[B25] Johnston A. (2017). “Perceiving visual time,” in *Routledge Handbook of the Philosophy of Temporal Experience*, ed. Phillips I. (Abingdon: Routledge), 275–286. 10.4324/9781315269641-22

[B26] Katyal S. He S. He B. Engel S. A. (2019). Frequency of alpha oscillation predicts individual differences in perceptual stability during binocular rivalry. *Hum. Brain Mapping* 40 2422–2433. 10.1002/hbm.24533 30702190 PMC6865672

[B27] Khoei M. A. Masson G. S. Perrinet L. U. (2013). Motion-based prediction explains the role of tracking in motion extrapolation. *J. Physiol. Paris* 107 409–420. 10.1016/j.jphysparis.2013.08.001 24036184

[B28] Khoei M. A. Masson G. S. Perrinet L. U. (2017). The flash-lag effect as a motion-based predictive shift. *PLoS Comput. Biol.* 13:e1005068. 10.1371/journal.pcbi.1005068 28125585 PMC5268412

[B29] Kleiner M. Brainard D. Pelli D. Ingling A. Murray R. Broussard C. (2007). What’s new in Psychtoolbox-3? *Perception* 36 1–16.

[B30] Kwon O.-S. Tadin D. Knill D. C. (2015). Unifying account of visual motion and position perception. *Proc. Natl. Acad. Sci. U.S.A.* 112 8142–8147. 10.1073/pnas.1500361112 26080410 PMC4491751

[B31] Levitt H. (1971). Transformed up-down methods in psychoacoustics. *J. Acoust. Soc. Am.* 49 467–477. 10.1121/1.1912375 5541744

[B32] Luna R. Serrano-Pedraza I. (2020). Evidence for different spatiotemporal mechanisms using duration thresholds: an individual differences approach. *Vis. Res.* 175 58–74. 10.1016/j.visres.2020.07.002 32712430

[B33] Makin T. R. Orban de Xivry J. J. (2019). Ten common statistical mistakes to watch out for when writing or reviewing a manuscript. *Elife* 8:e48175. 10.7554/eLife.48175 31596231 PMC6785265

[B34] McGraw P. V. Whitaker D. Skillen J. Chung S. T. (2002). Motion adaptation distorts perceived visual position. *Curr. Biol.* 12 2042–2047. 10.1016/s0960-9822(02)01354-4 12477394

[B35] Melnick M. D. Harrison B. R. Park S. Bennetto L. Tadin D. (2013). A strong interactive link between sensory discriminations and intelligence. *Curr. Biol.* 23 1013–1017. 10.1016/j.cub.2013.04.053 23707433 PMC3702042

[B36] Milton A. Pleydell-Pearce C. W. (2016). The phase of pre-stimulus alpha oscillations influences the visual perception of stimulus timing. *Neuroimage* 133 53–61. 10.1016/j.neuroimage.2016.02.065 26924284 PMC4907635

[B37] Minami S. Amano K. (2017). Illusory jitter perceived at the frequency of alpha oscillations. *Curr. Biol.* 27 2344–2351. e2344. 10.1016/j.cub.2017.06.033 28756954

[B38] Mollon J. D. Bosten J. M. Peterzell D. H. Webster M. A. (2017). Individual differences in visual science: what can be learned and what is good experimental practice? *Vis. Res.* 141 4–15. 10.1016/j.visres.2017.11.001 29129731 PMC5730466

[B39] Nijhawan R. (2002). Neural delays, visual motion and the flash-lag effect. *Trends Cogn. Sci.* 6 387–393. 10.1016/s1364-6613(02)01963-0 12200181

[B40] Nishida S. Y. Johnston A. (1999). Influence of motion signals on the perceived position of spatial pattern. *Nature* 397 610–612. 10.1038/17600 10050853

[B41] Perrinet L. U. Masson G. S. (2012). Motion-based prediction is sufficient to solve the aperture problem. *Neural Comput.* 24 2726–2750. 10.1162/NECO_a_00332 22734489 PMC3472550

[B42] Peterzell D. H. Kennedy J. F. (2016). Discovering sensory processes using individual differences: a review and factor analytic manifesto. *Electron. Imaging* 2016 1–11. 10.2352/issn.2470-1173.2016.16.hvei-112

[B43] Roach N. W. McGraw P. V. Johnston A. (2011). Visual motion induces a forward prediction of spatial pattern. *Curr. Biol.* 21 740–745. 10.1016/j.cub.2011.03.031 21514158 PMC3093611

[B44] Samaha J. Postle B. R. (2015). The speed of alpha-band oscillations predicts the temporal resolution of visual perception. *Curr. Biol.* 25 2985–2990. 10.1016/j.cub.2015.10.007 26526370 PMC4654641

[B45] Scarfe P. Johnston A. (2011). Global motion coherence can influence the representation of ambiguous local motion. *J. Vis.* 11:6. 10.1167/11.12.6 21997477

[B46] Senior C. Ward J. David A. S. (2002). Representational momentum and the brain: an investigation into the functional necessity of V5/MT. *Vis. Cogn.* 9 81–92. 10.1080/13506280143000331

[B47] Snowden R. J. (1998). Shifts in perceived position following adaptation to visual motion. *Curr. Biol.* 8 1343–1345. 10.1016/s0960-9822(07)00567-2 9843685

[B48] Tadin D. Park W. J. Dieter K. C. Melnick M. D. Lappin J. S. Blake R. (2019). Spatial suppression promotes rapid figure-ground segmentation of moving objects. *Nat. Commun.* 10 1–12. 10.1038/s41467-019-10653-8 31266956 PMC6606582

[B49] Thompson P. (1982). Perceived rate of movement depends on contrast. *Vis. Res.* 22 377–380. 10.1016/0042-6989(82)90153-5 7090191

[B50] Valera F. J. Toro A. John E. R. Schwartz E. L. (1981). Perceptual framing and cortical alpha rhythm. *Neuropsychologia* 19 675–686. 10.1016/0028-3932(81)90005-1 7312152

[B51] VanRullen R. (2016). Perceptual cycles. *Trends Cogn. Sci.* 20 723–735. 10.2466/pms.1980.50.3.723 27567317

[B52] VanRullen R. Dubois J. (2011). The psychophysics of brain rhythms. *Front. Psychol.* 2:203. 10.3389/fpsyg.2011.00203 21904532 PMC3163286

[B53] Whitaker D. McGraw P. V. Pearson S. (1999). Non-veridical size perception of expanding and contracting objects. *Vis. Res.* 39 2999–3009. 10.1016/s0042-6989(99)00010-3 10664799

[B54] Wilmer J. B. Nakayama K. (2007). Two distinct visual motion mechanisms for smooth pursuit: evidence from individual differences. *Neuron* 54 987–1000. 10.1016/j.neuron.2007.06.007 17582337 PMC2562445

