# Supplementary material for: Exploring the Common Mechanisms of Motion-Based Visual Prediction
Source: Front Psychol. 2022 Mar 22;13:827029. doi: 10.3389/fpsyg.2022.827029 (PMC8981589; doi:10.3389/fpsyg.2022.827029)

**Supplementary Material**

**Supplementary Material 1.** Minimum motion task for MISC

We used the program ‘MinimumMotionExp’ (Cavanagh et al., 1987) in PTB-3, to measure the perceptually equiluminant point for green relative to the maximum luminance red (the maximum luminance that the monitor could generate for red). As the stimulus size or eccentricity might affect the result, we employed two sizes of annular stimuli (inter-/outer- diameters were 0/2 and 11/15 deg, respectively) centred on fixation, and averaged the results to determine the final iso-luminant point. There were five repetitions for each stimulus size condition, leading to 10 trials for a single test run. Participants practised for 1-4 runs before the formal test run.

**Reference:** Cavanagh, P., MacLeod, D. I., & Anstis, S. M. (1987). Equiluminance: spatial and temporal factors and the contribution of blue-sensitive cones. *JOSA A*, *4*(8), 1428-1438.

**Supplementary Material 2.** Participant performance for all measures

| **Participant** | **MISC** | | **MIPS** | **AISS** | **SMT** | |
| --- | --- | --- | --- | --- | --- | --- |
|  | **PSE**  **(Hz)** | **Slope** | **B1**  **(retinal deg/s)** | **B1**  **(angular deg/s)** | **PSE**  **(Hz)** | **Slope** |
| **1** | 10.44 | 1.48 | 2.75** | 0.57* | 6.93 | 0.81 |
| **2** | 8.62 | 1.48 | 2.45* | 1.11* | 6.40 | 0.30 |
| **3** | 10.52 | 0.75 | 3.60** | 3.46** | 7.02 | 0.37 |
| **4** | 10.75 | 0.36 | 3.27** | 1.20* | 5.58 | 0.29 |
| **5** | 9.85 | 1.34 | 1.68** | 1.17* | 5.63 | 0.49 |
| **6** | 8.86 | 0.91 | 0.83 | 1.83** | 6.73 | 0.49 |
| **7** | 8.92 | 1.85 | 3.25** | 1.15** | 6.78 | 0.46 |
| **8** | 10.24 | 0.41 | 3.51** | 2.10* | 6.71 | 0.54 |
| **9** | 9.26 | 1.03 | 2.63* | 0.23 | 6.72 | 0.35 |
| **10** | 10.02 | 1.34 | 2.54* | -0.74 | 6.73 | 0.19 |
| **11** | 10.73 | 0.87 | 5.37** | 1.67 | 7.08 | 0.28 |
| **12** | 10.68 | 2.07 | 3.59** | 0.43 | 6.29 | 0.43 |
| **13** | 8.51 | 0.83 | 3.28** | 1.13** | 6.77 | 0.51 |
| **14** | 8.36 | 1.32 | 2.19* | 0.40 | 6.95 | 0.49 |
| **15** | 8.20 | 1.70 | 2.26* | 0.42 | 6.54 | 0.39 |
| **16** | 8.26 | 0.54 | 2.62** | 1.65 | 5.78 | 0.92 |
| **17** | 10.49 | 1.52 | 2.49* | 0.54 | 5.88 | 0.14 |
| **18** | 8.97 | 1.85 | 1.22 | -0.45 | 7.40 | 0.41 |
| **19** | 8.24 | 0.70 | 1.23* | 1.15* | 6.50 | 0.34 |
| **20** | 9.55 | 1.01 | 2.72* | 1.98** | 5.66 | 0.57 |
| **21** | 8.73 | 1.39 | 1.31* | 0.45 | 6.84 | 0.31 |
| **22** | 8.93 | 0.91 | 2.67** | 1.51 | 5.54 | 0.74 |
| **23** | 8.53 | 1.76 | 3.24** | 0.88** | 6.60 | 0.49 |
| **24** | 10.40 | 0.63 | 2.78* | 0.66 | 6.87 | 0.53 |
| **25** | 9.37 | 1.10 | 1.98** | 0.51 | 6.31 | 0.28 |
| **26** | 8.42 | 0.59 | 3.41* | 0.28 | 5.93 | 0.38 |
| **27** | 8.15 | 0.51 | 2.61* | 1.77* | 5.89 | 0.44 |
| **28** | 9.99 | 1.21 | 3.30** | 1.15* | 6.71 | 0.56 |

*Note.* * *p* <= .05; ** *p* < .01; see also Figure 1 and Figure 2.

**Supplementary Material 3.** Each participant’s performance in the MIPS task. The illusory positional shift (retinal degree) increased as the stimuli duration increased from 16 to 83 ms (the first five circles in each figure) in the direction of motion. The mean and standard deviation for each duration condition (calculated from at least 12 reversals) is shown in each figure.


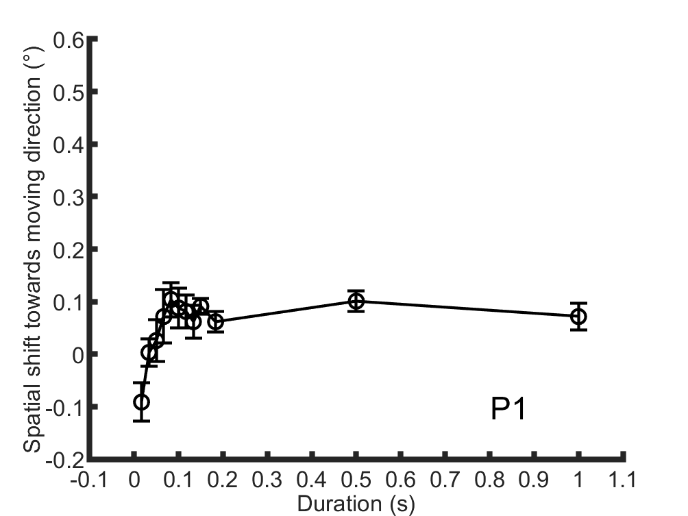

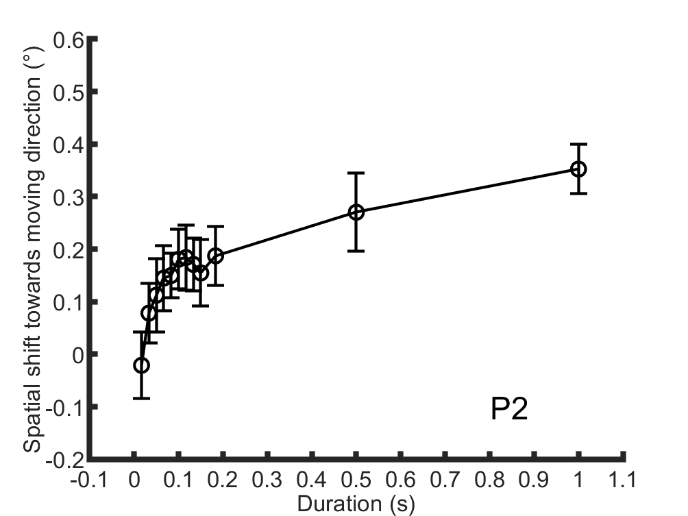


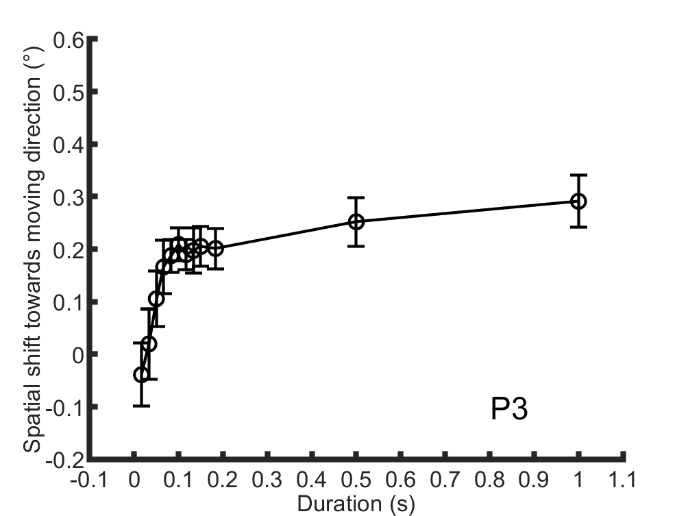

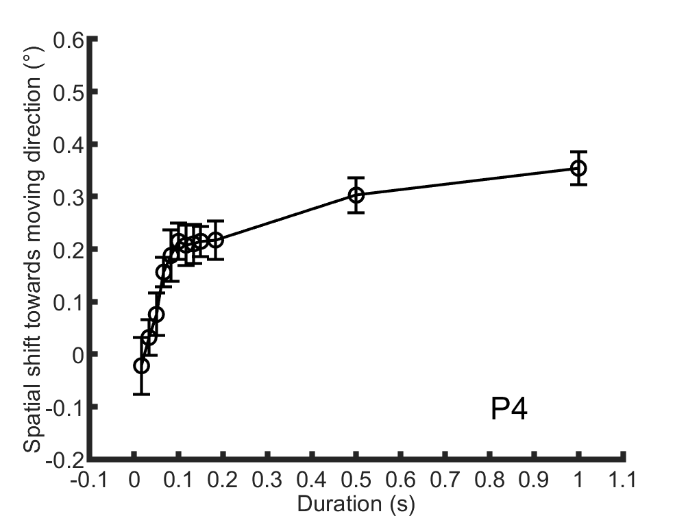


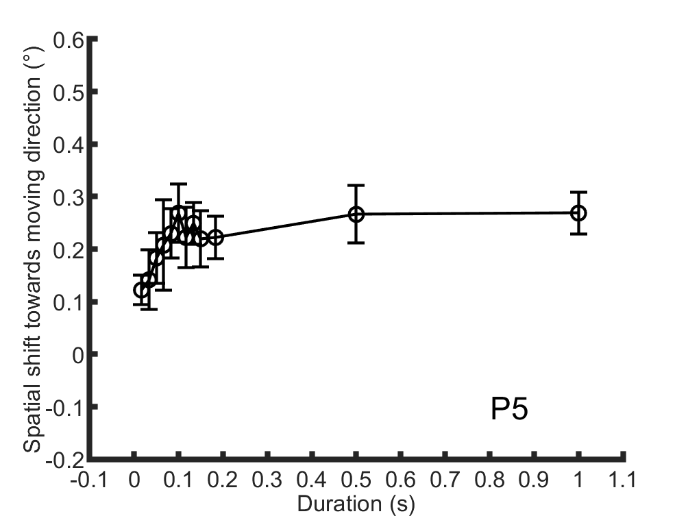

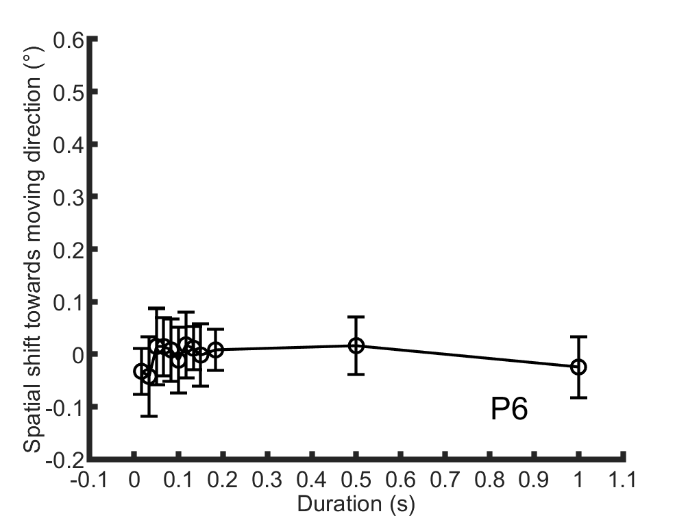


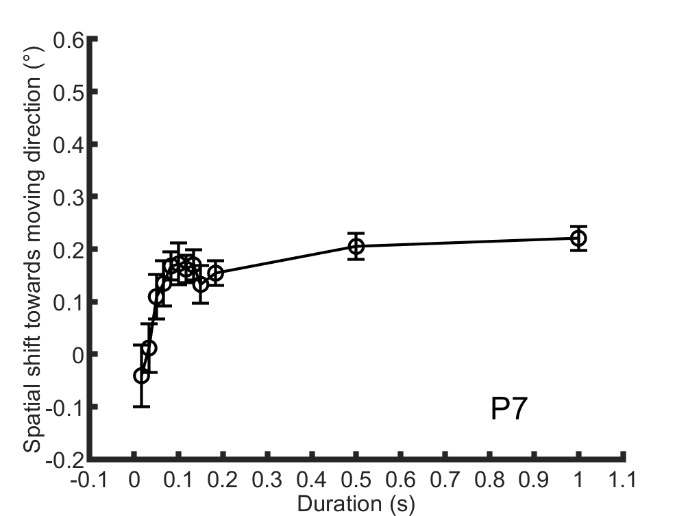

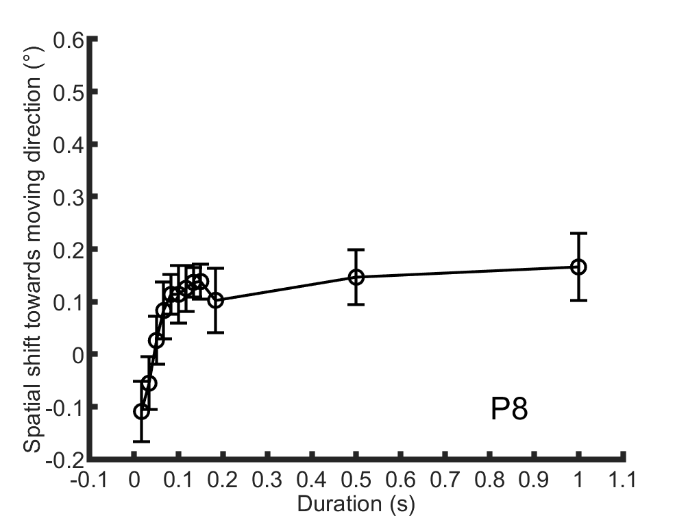


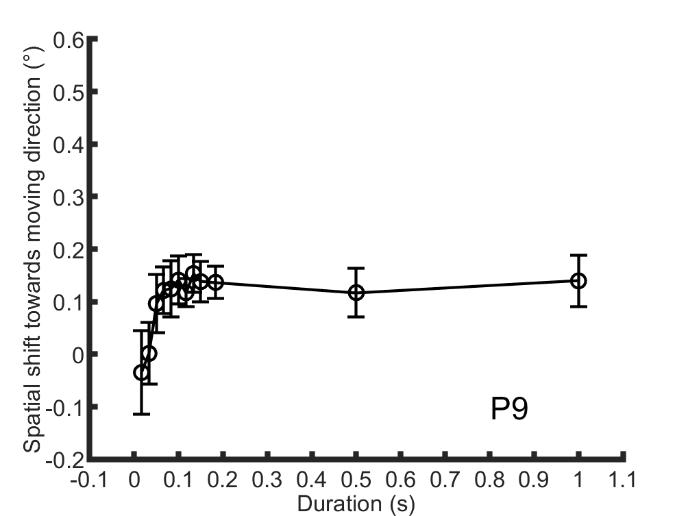

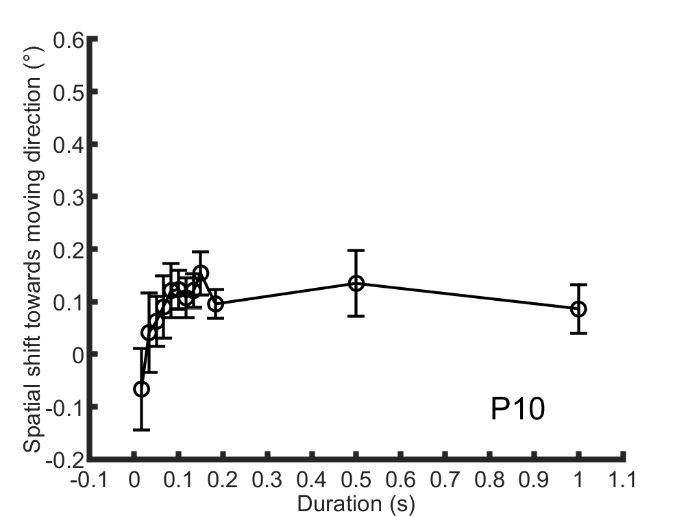


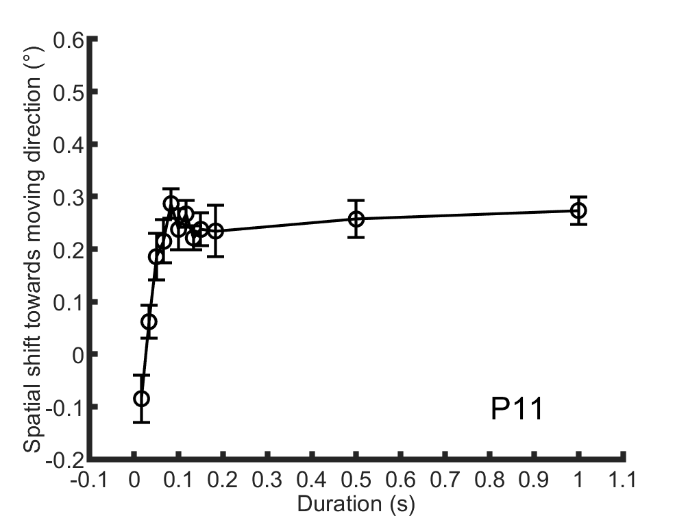

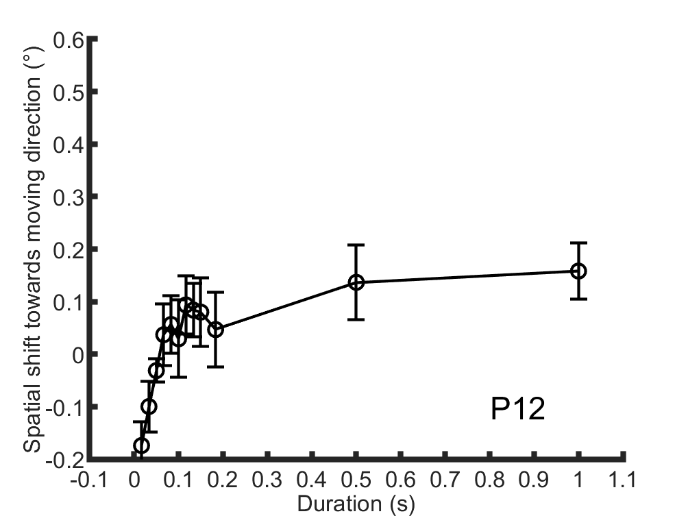


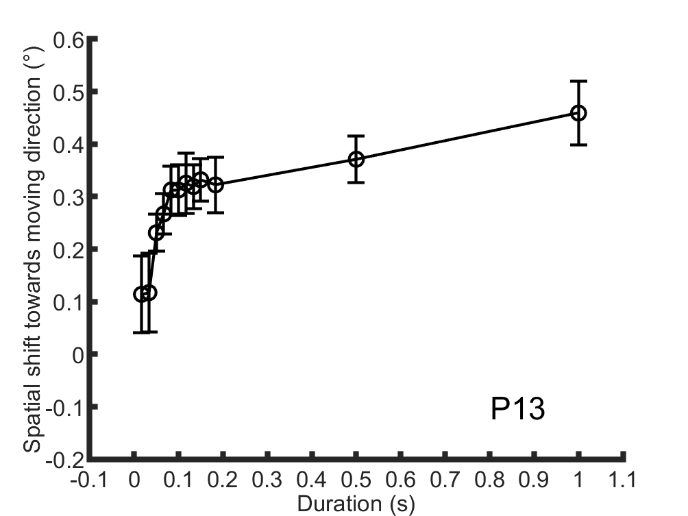

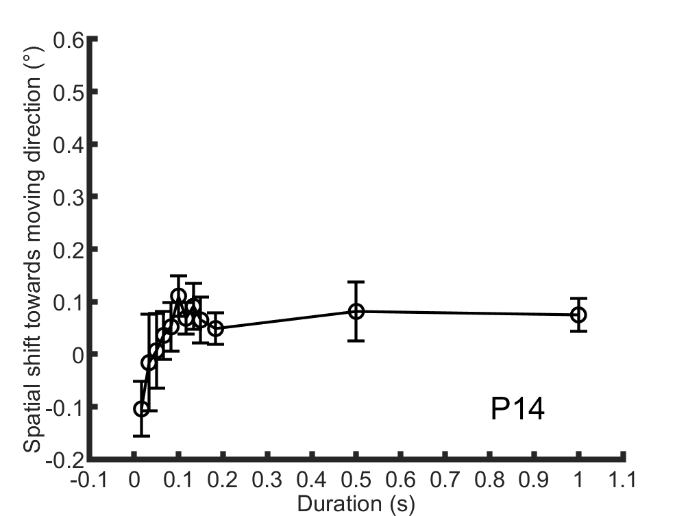


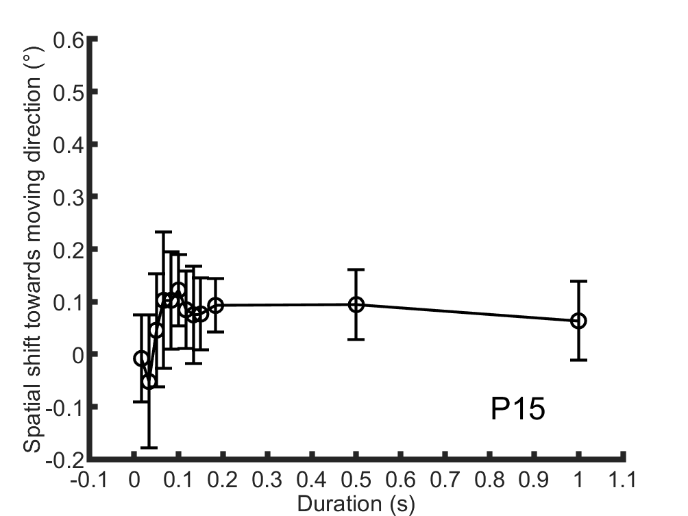

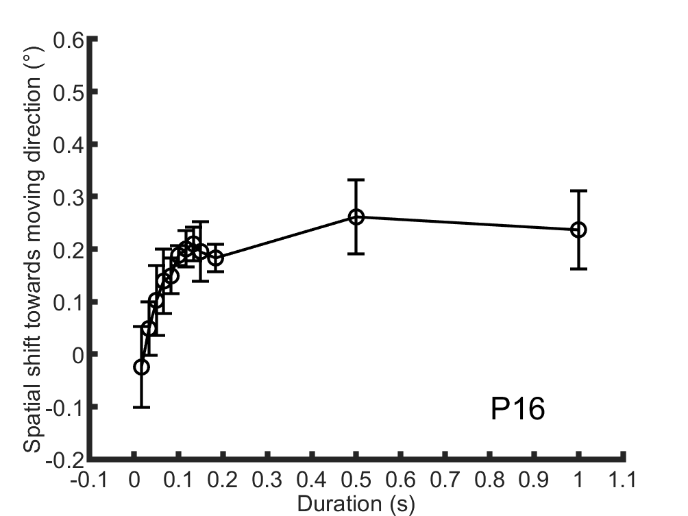


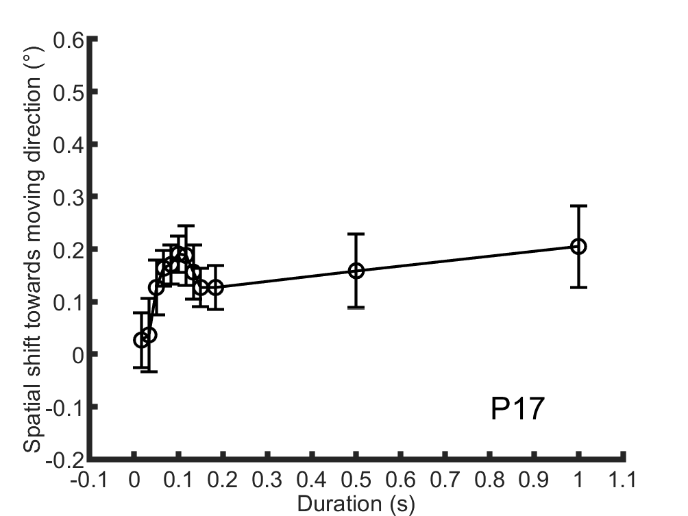

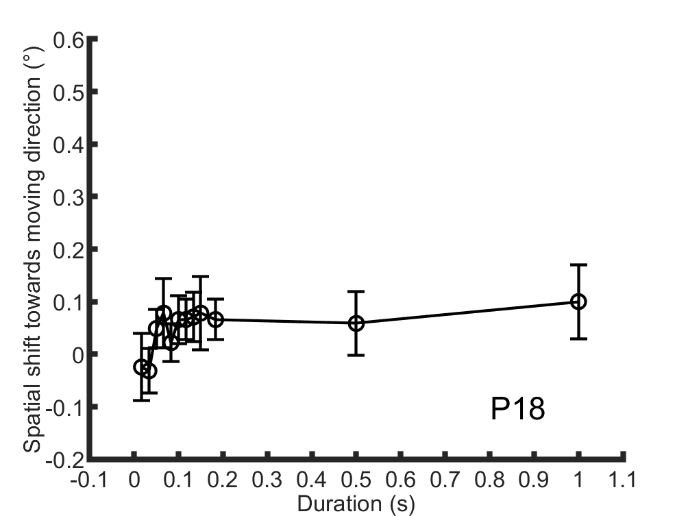


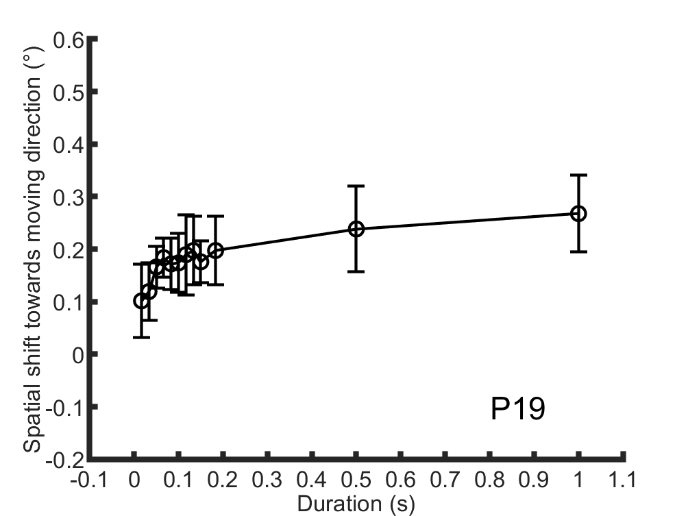

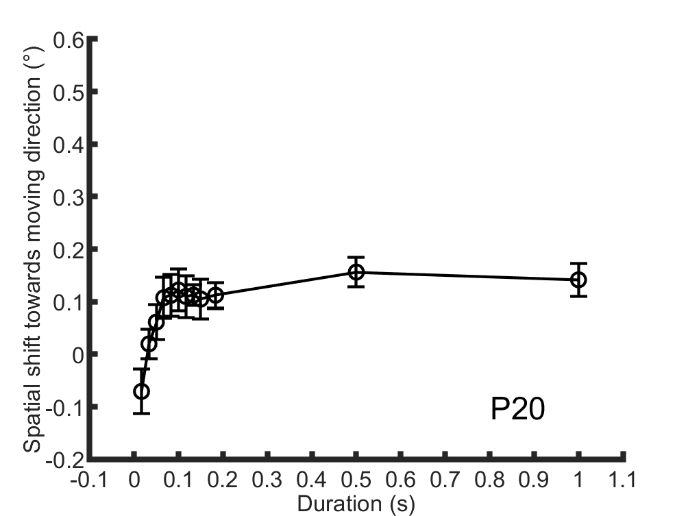

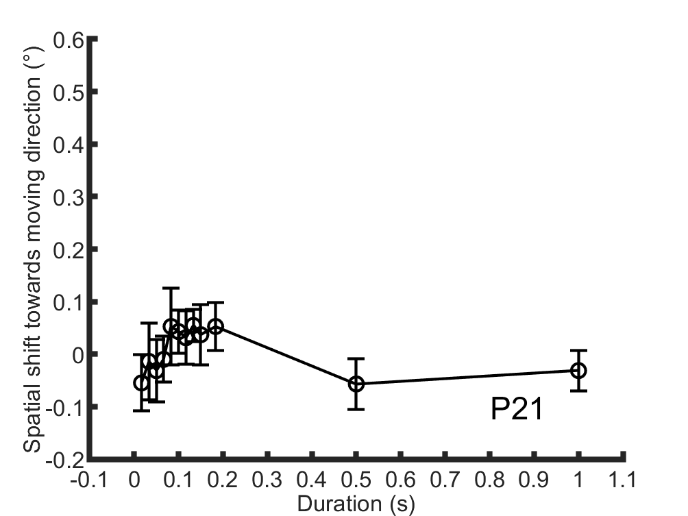

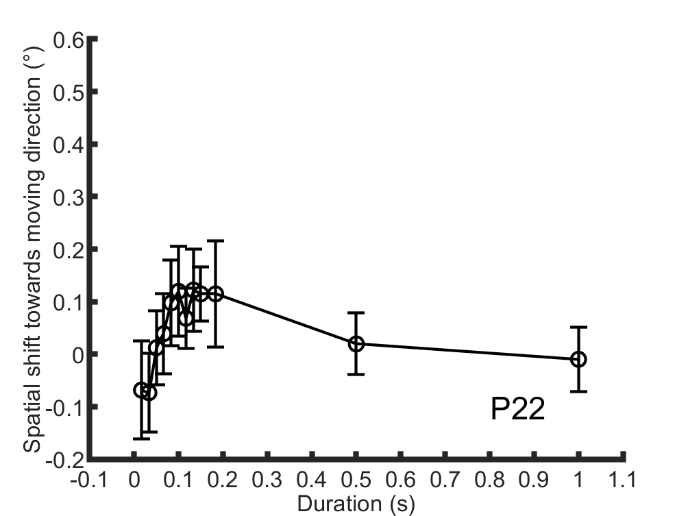


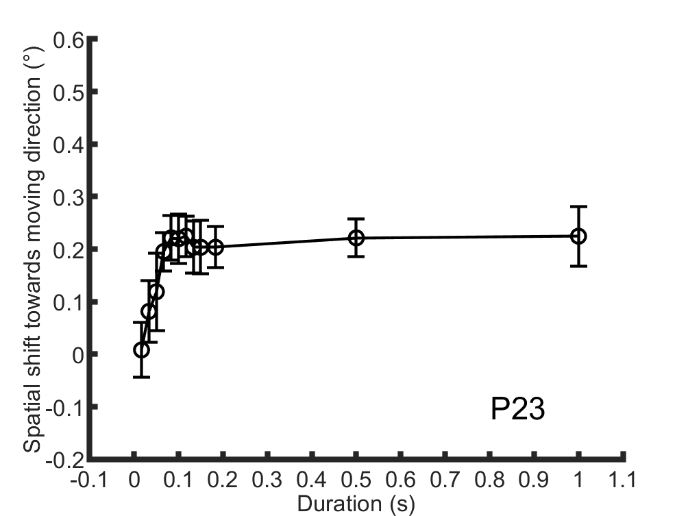

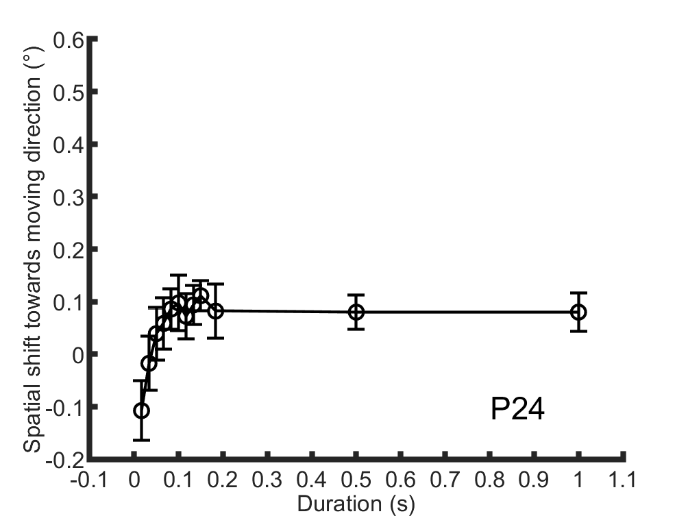


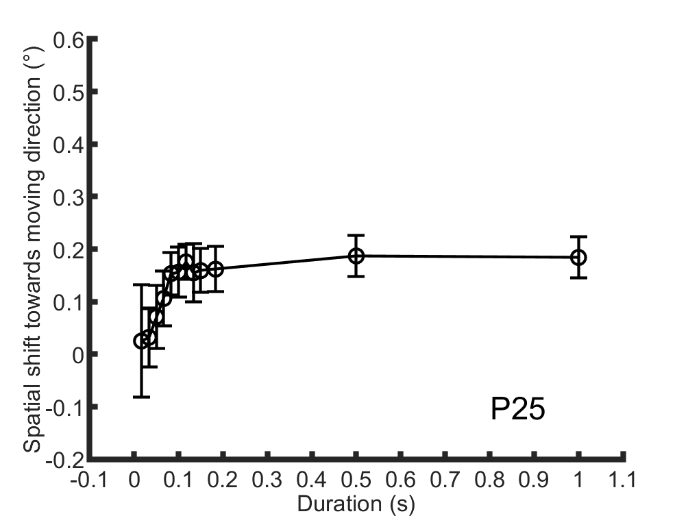

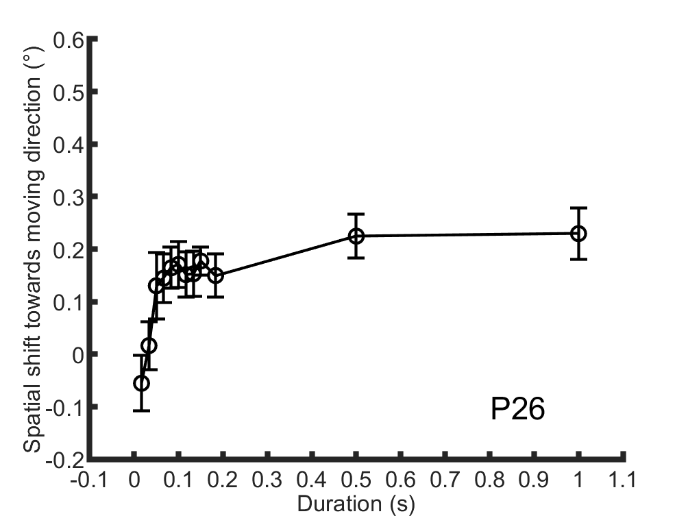


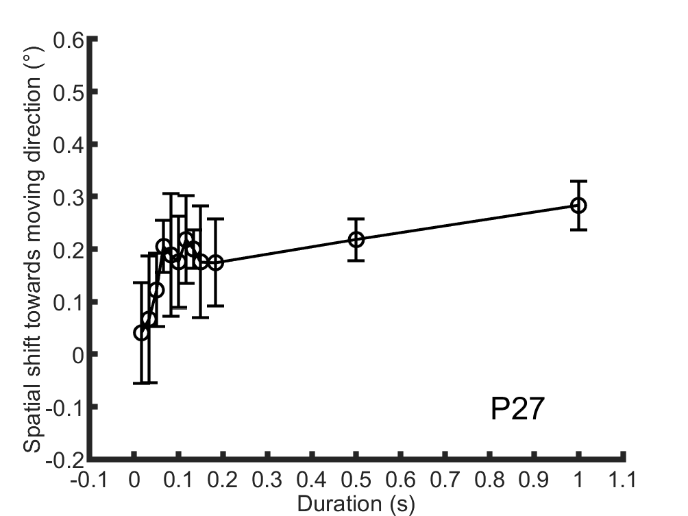

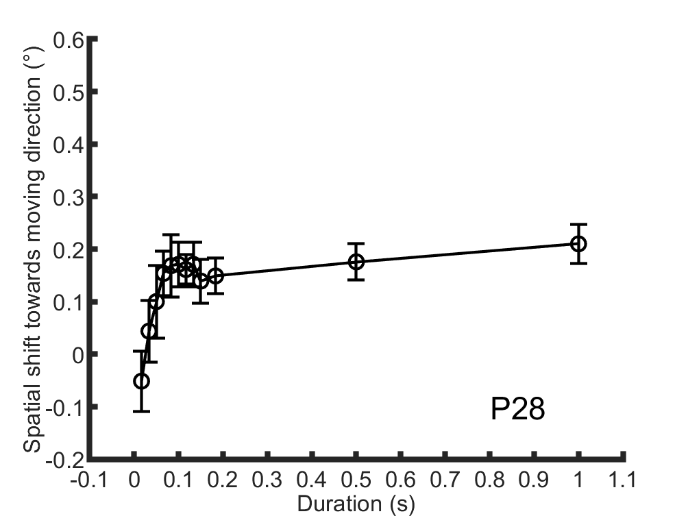


**Supplementary Material 4.** Each participant’s performance in the AISS task. The illusory spatial shift towards the MAE direction (angular degree) accumulated as the T-C interval (s) increased. The mean and standard deviation on each condition (calculated from at least 12 reversals) is shown in each figure.


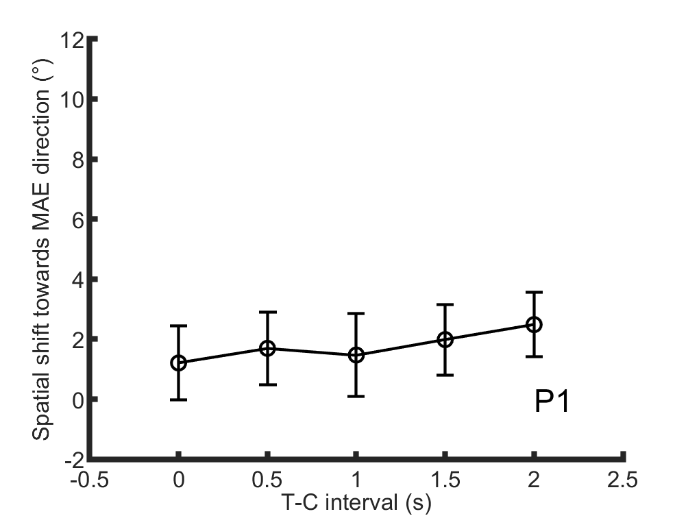

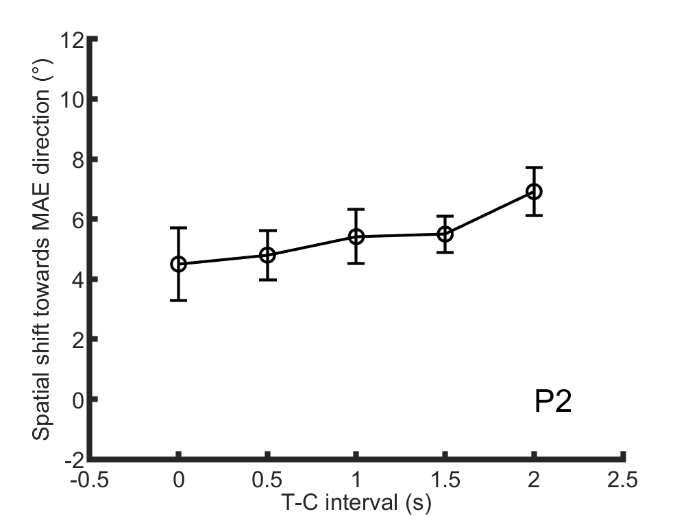


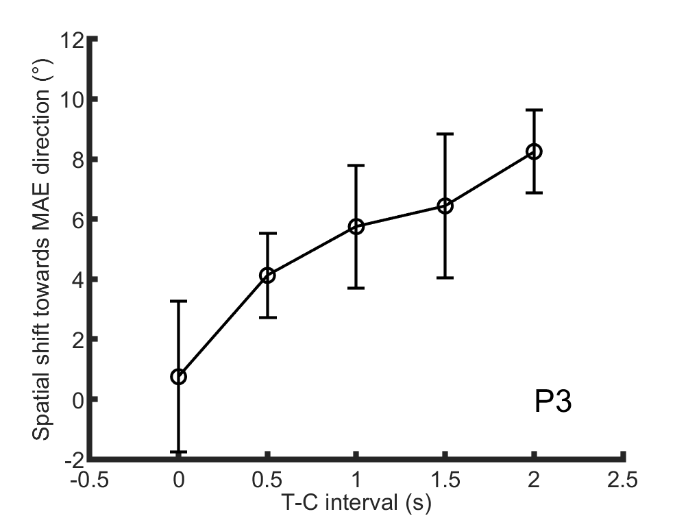

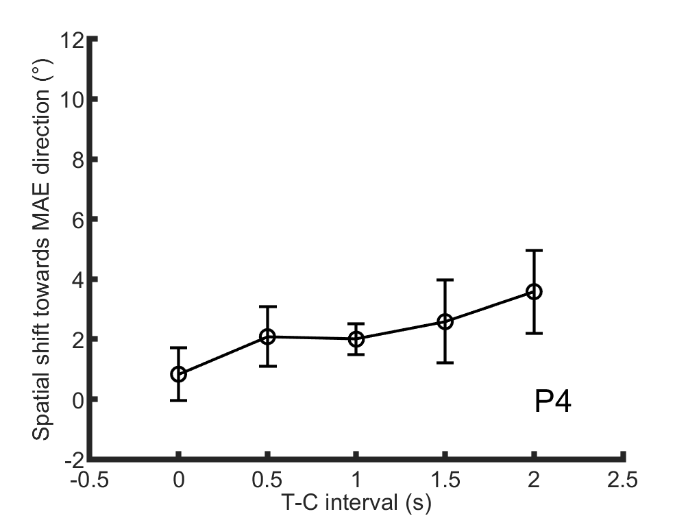


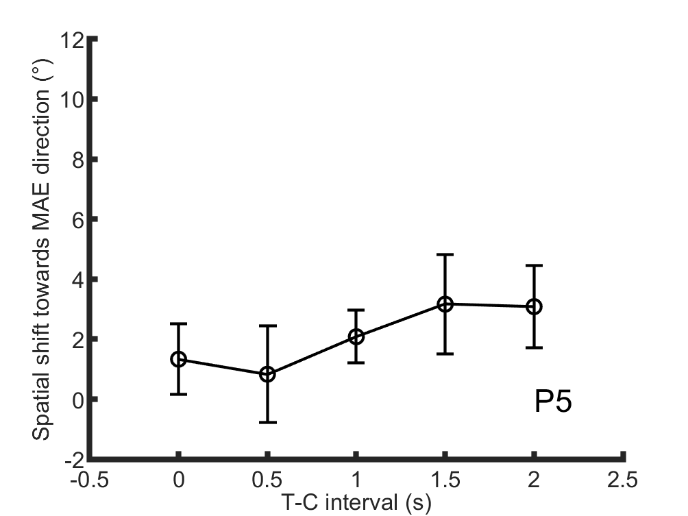

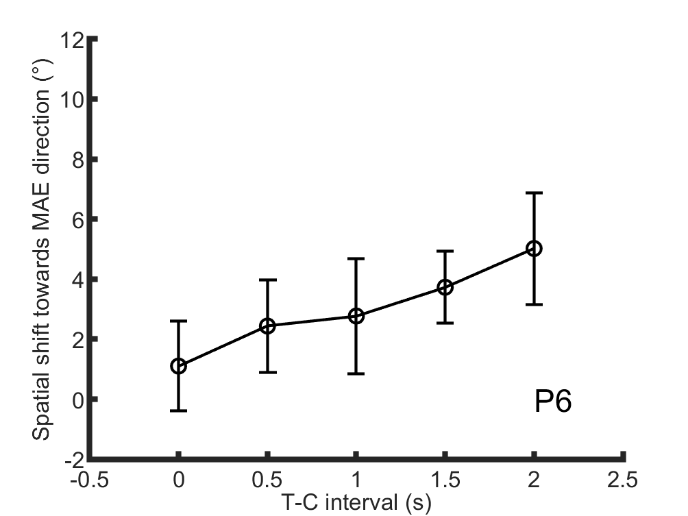


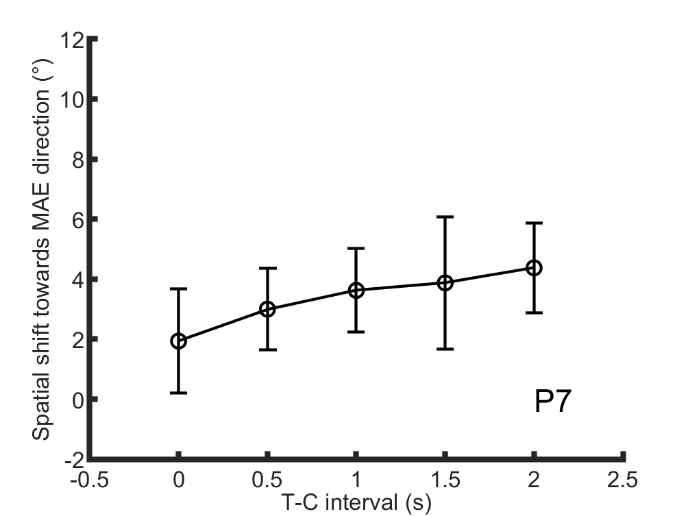

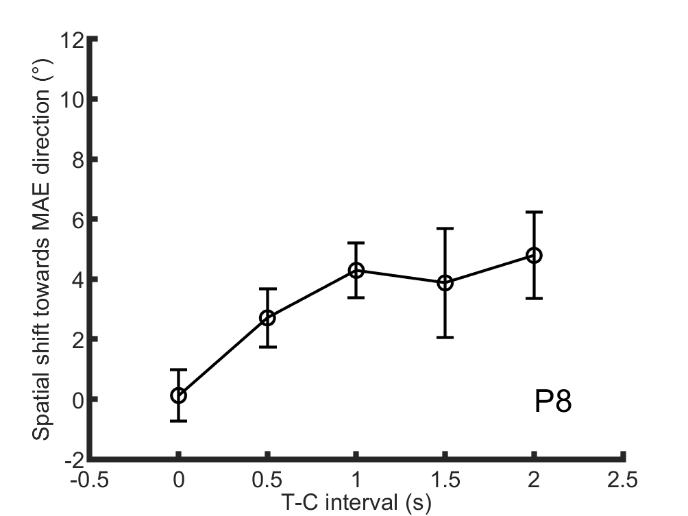


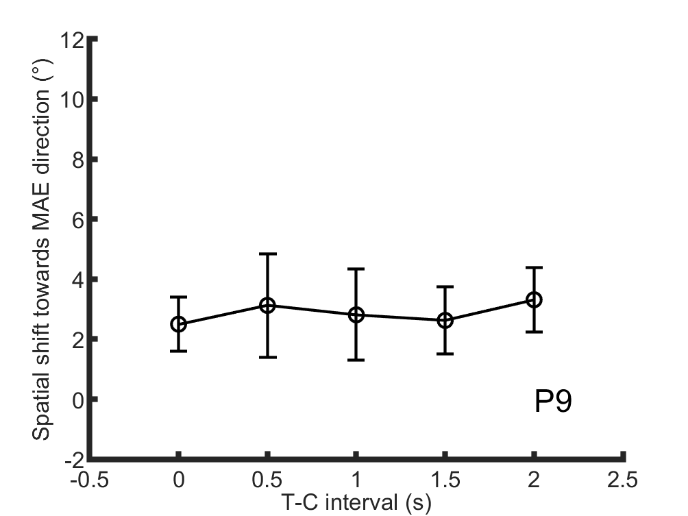

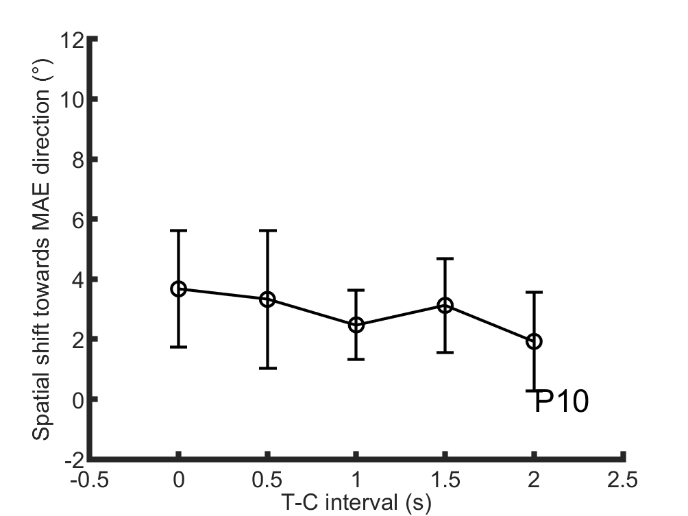


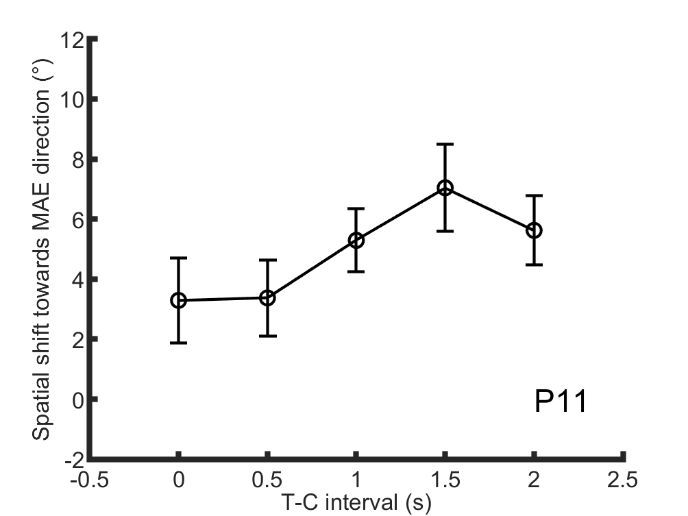

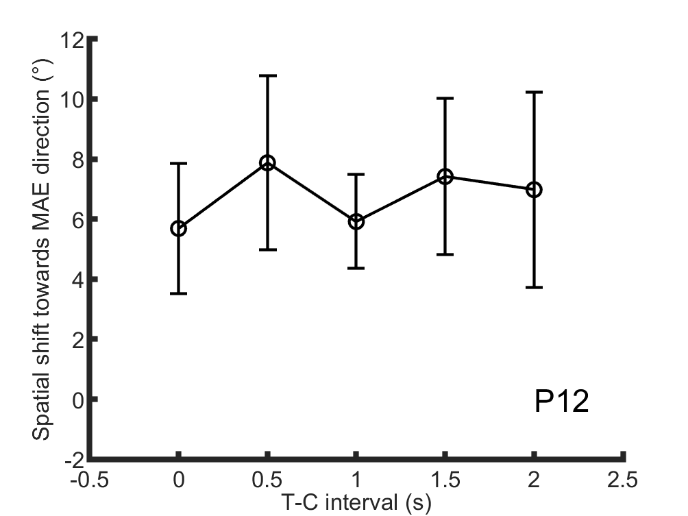

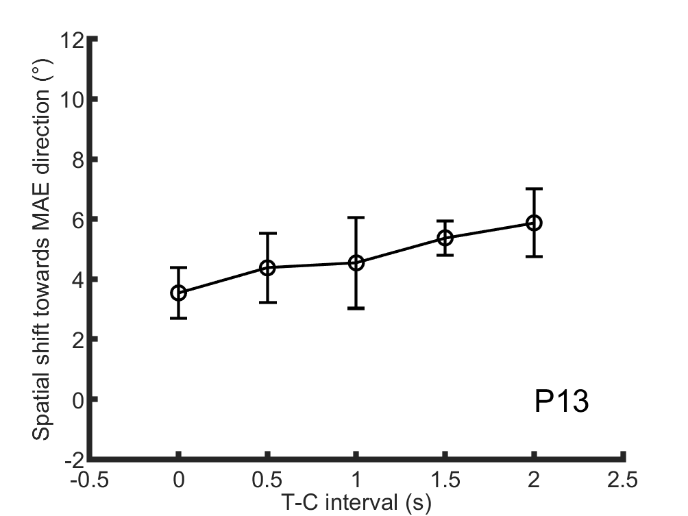

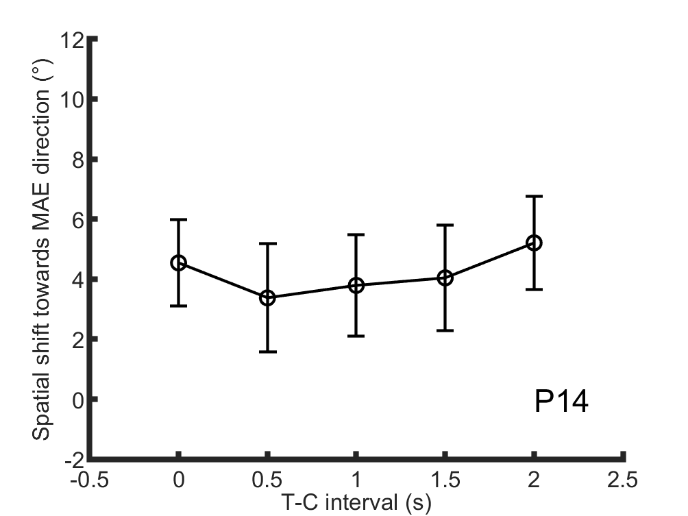


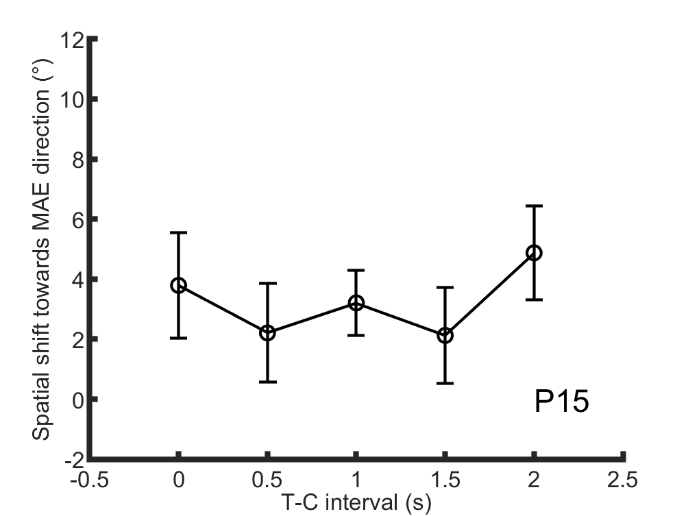

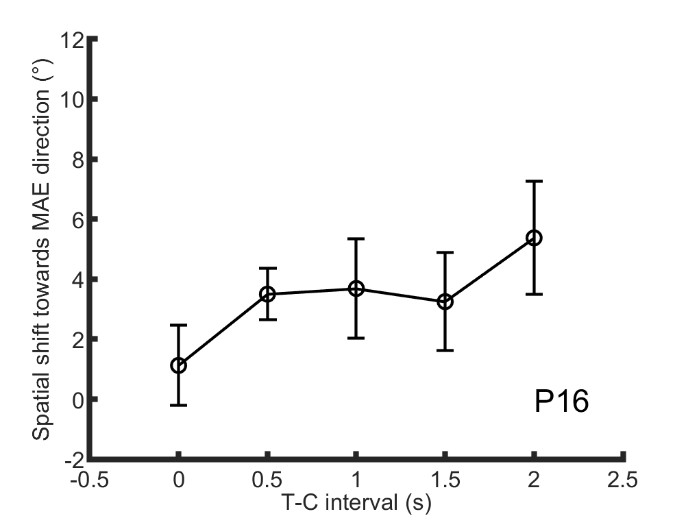


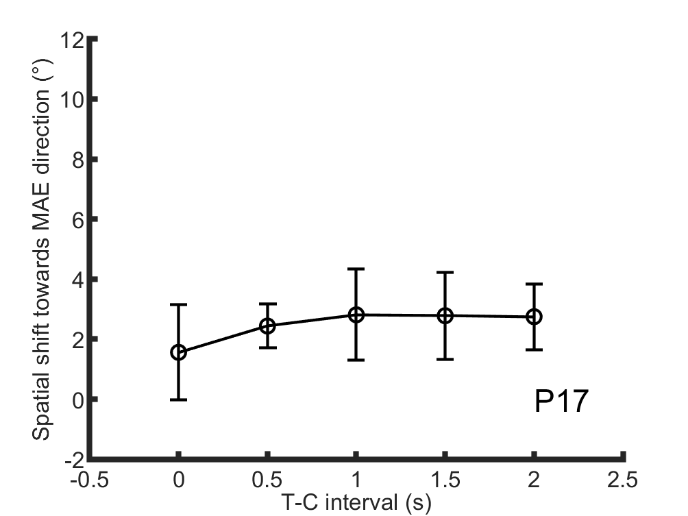

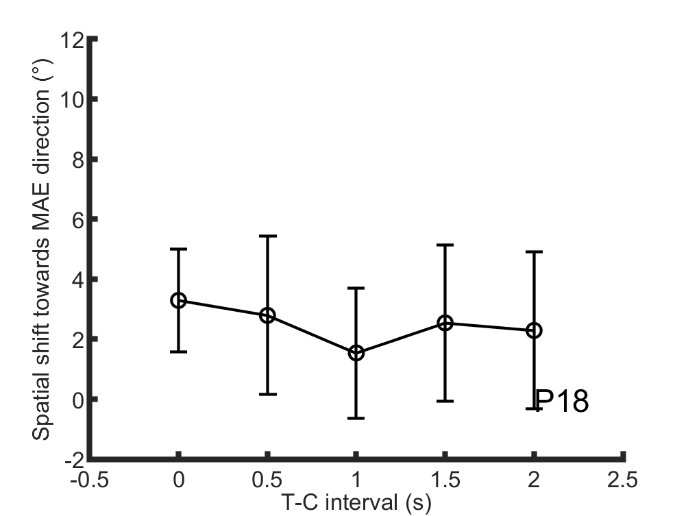


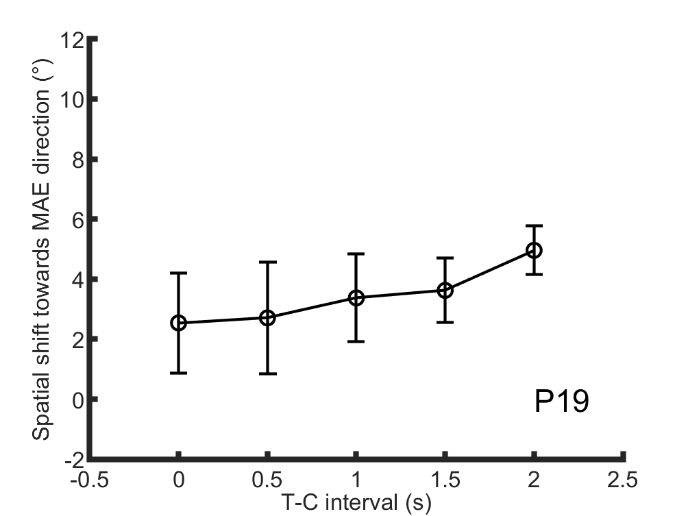

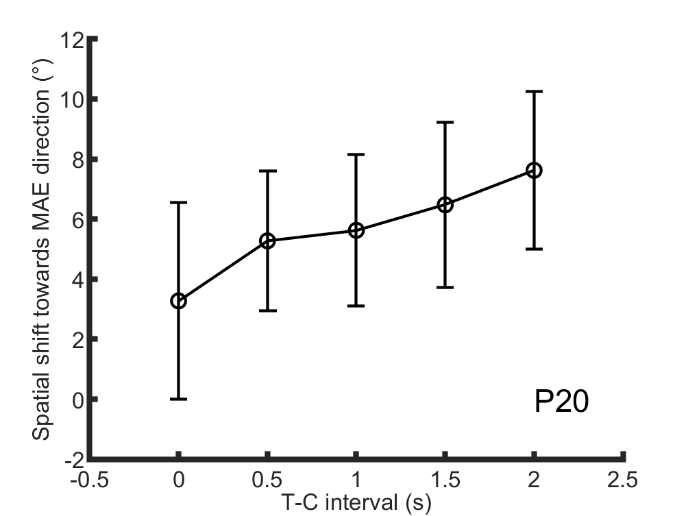


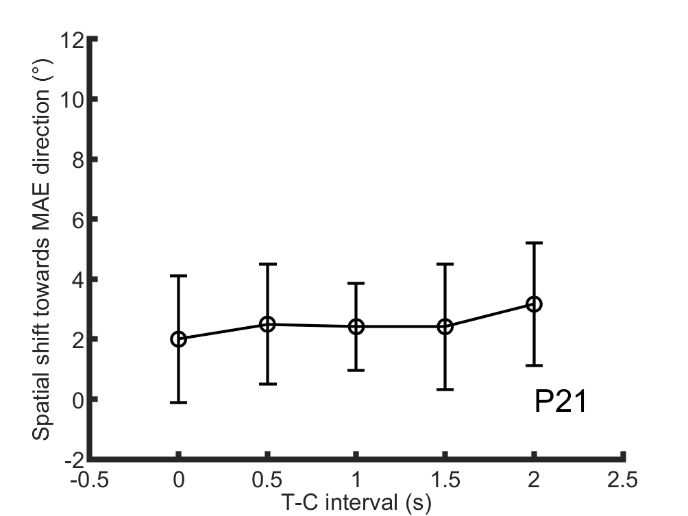

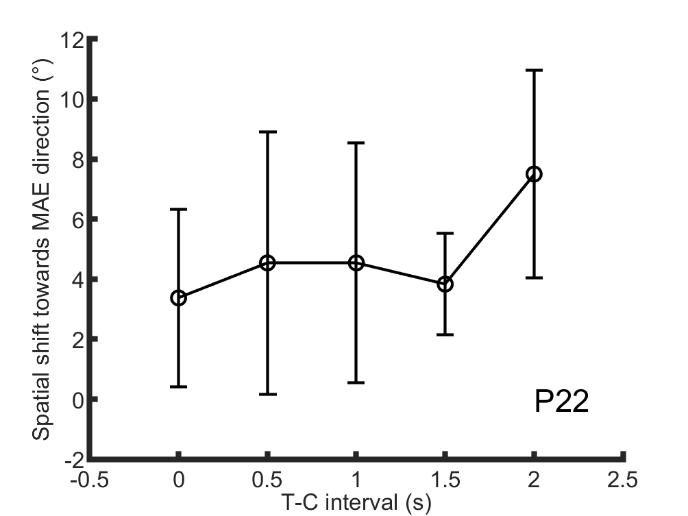


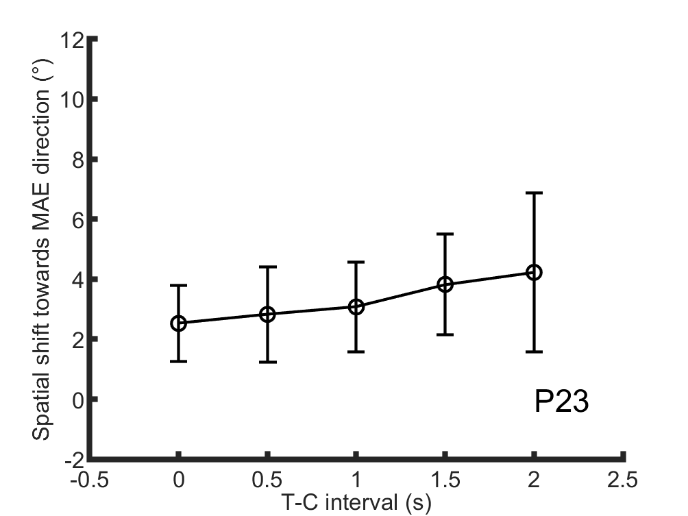

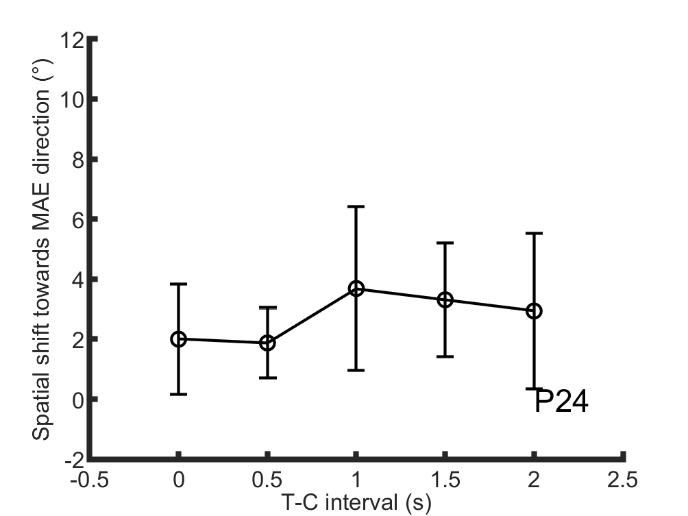


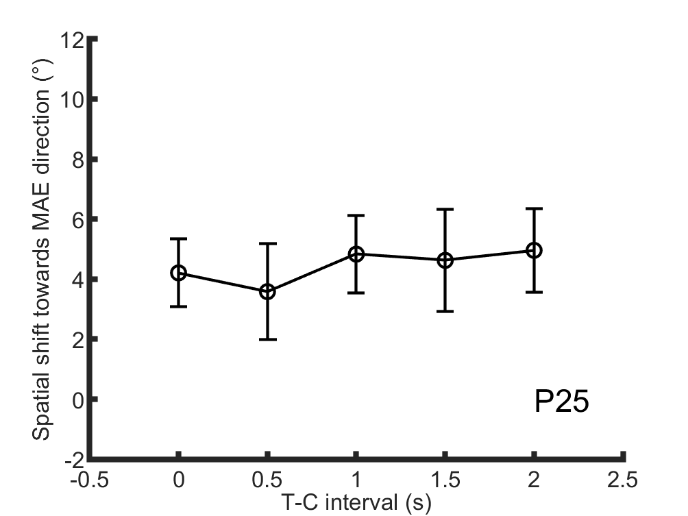

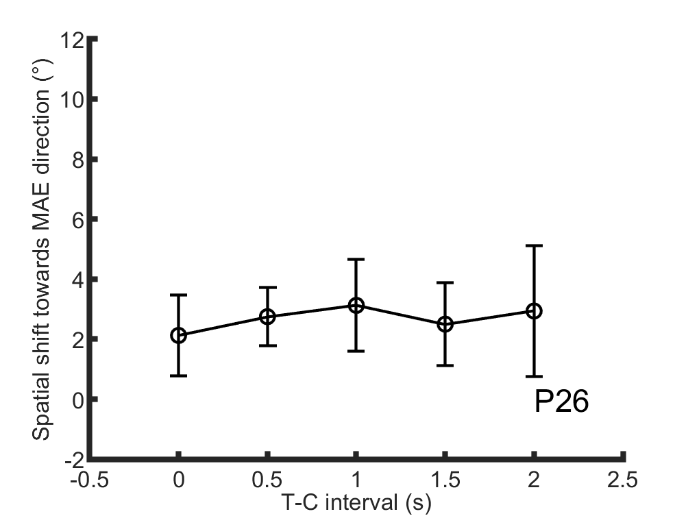


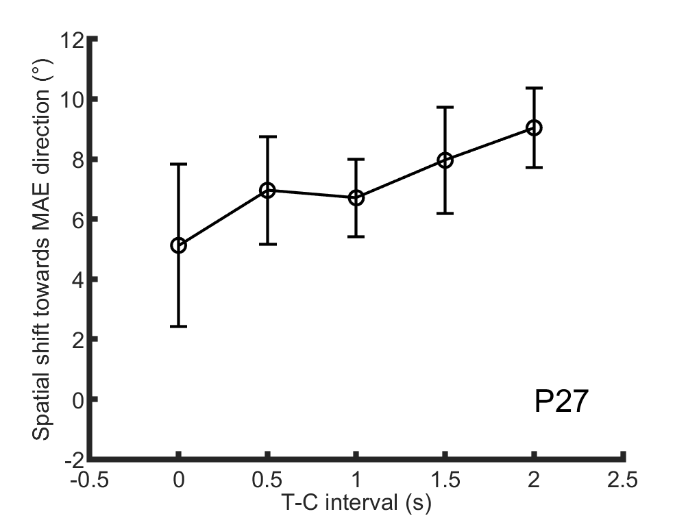

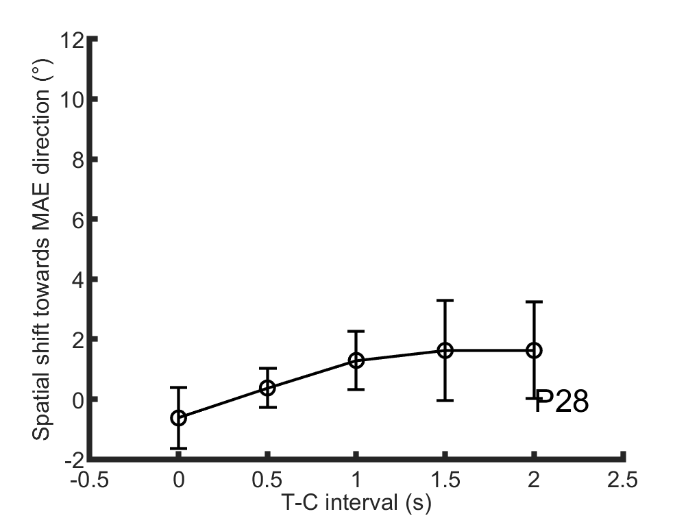


**Supplementary Material 5.** Scatter plots of participant performance data for all pairings of the four tasks, using all the participant data (N = 28). Spearman’s *r* and *p*-value calculated from each paired data set are shown in each figure. (A) The plot of MISC and MIPS scores. The illusory jitter frequency in MISC and accrual rate in MIPS were moderately correlated. There seems to be an outlier for the MIPS data (5.37 °/s, on the figure’s top-right corner). Although, Spearman’s correlation is rank-based and has an advantage of the robustness to outliers, we also conducted the correlation analysis while excluding this outlier. The result remained significant (Spearman’s *r* = 0.41, *p* = 0.04, N = 27). (B) The plot of MISC and AISS scores. (C) The plot of MISC and SMT scores. (D) The plot of MIPS and AISS scores. (E) The plot of MIPS and SMT scores. (F) The plot of AISS and SMT scores. All correlations in (B-F) were non-significant.

**A B**


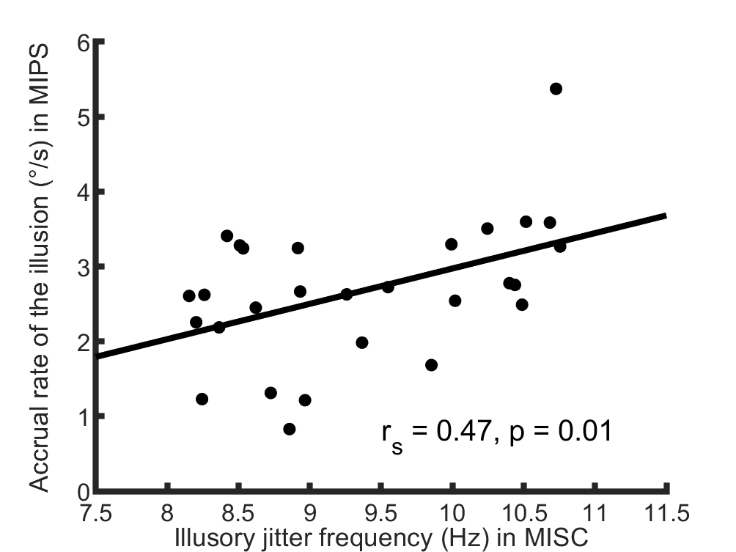

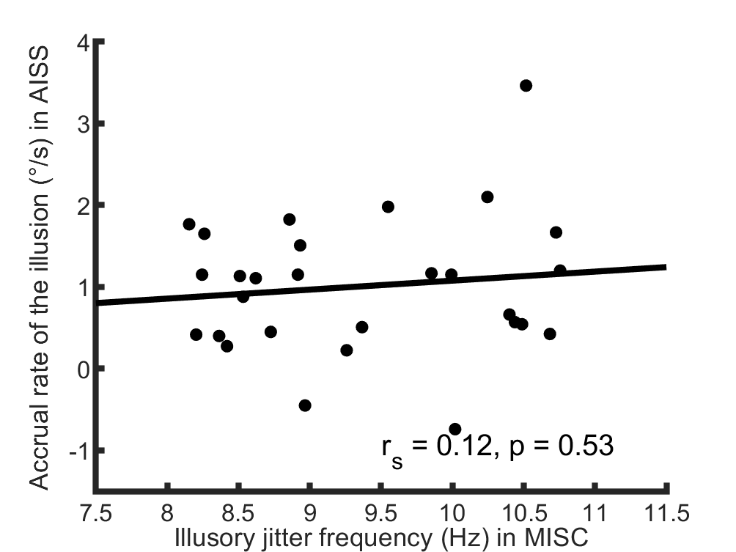


**C D**


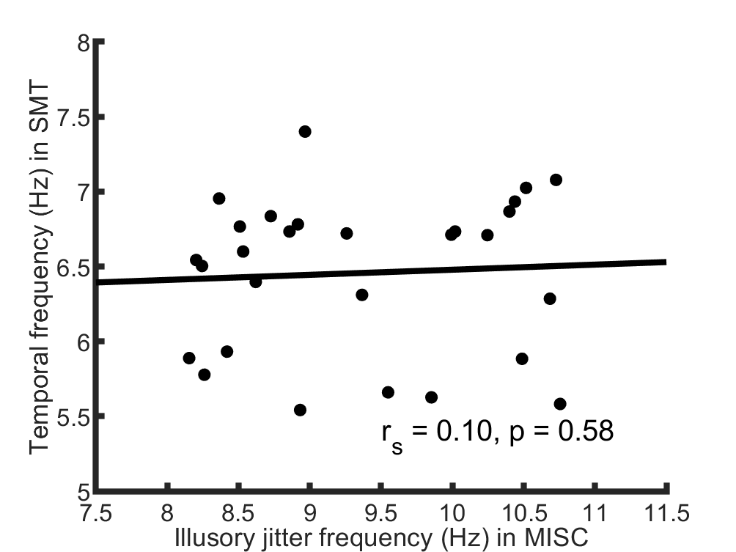

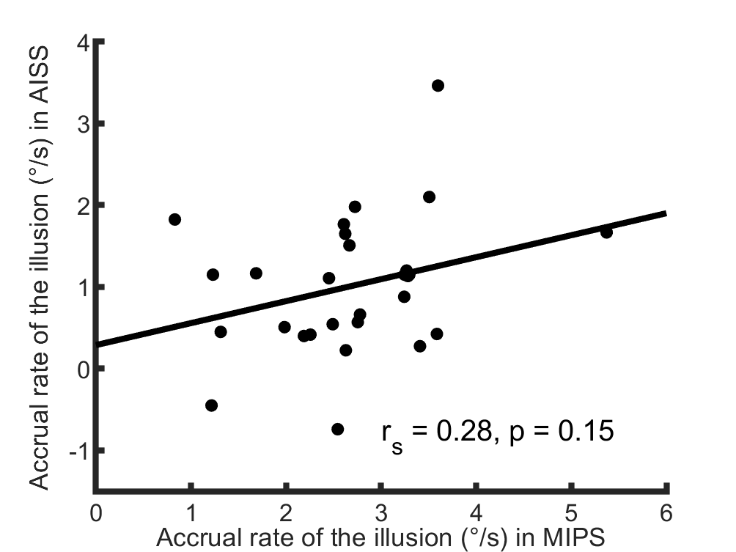


**E F**


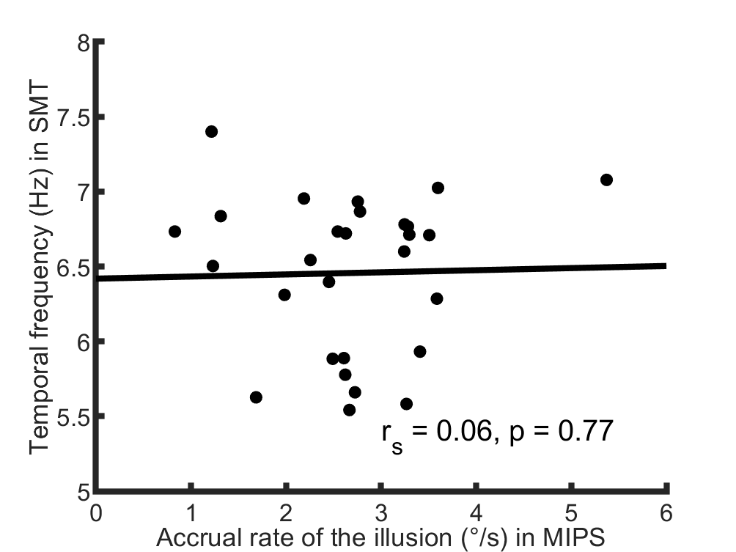

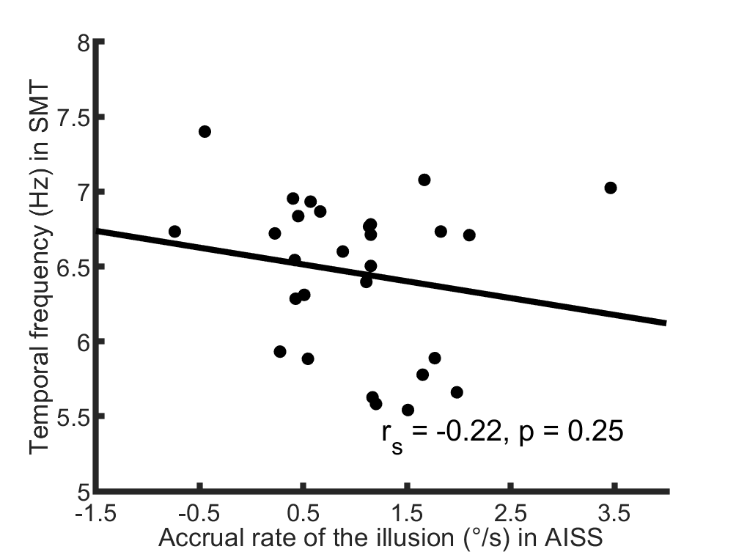


**Supplementary Material 6.** All histograms of Spearman’s *r* and *p*-values for the bootstrapping result.


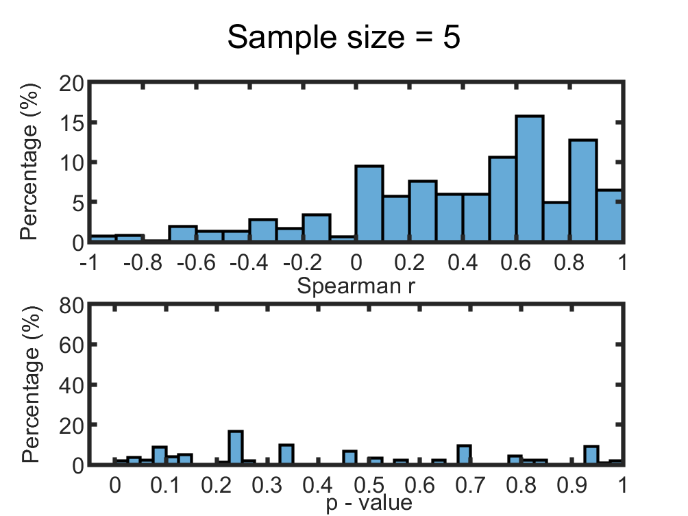

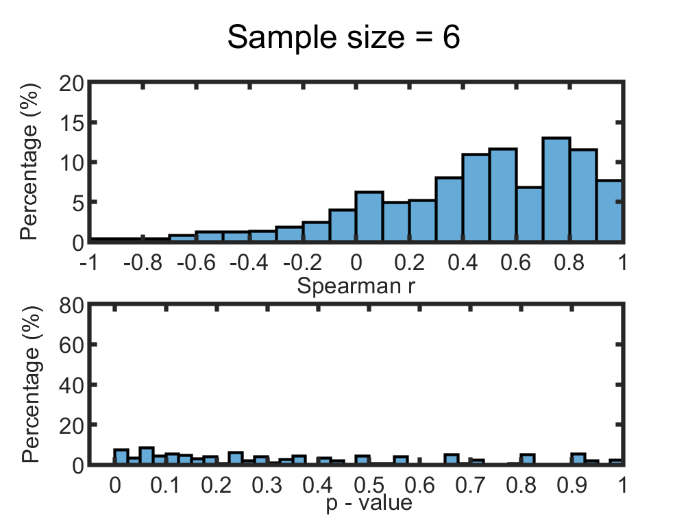


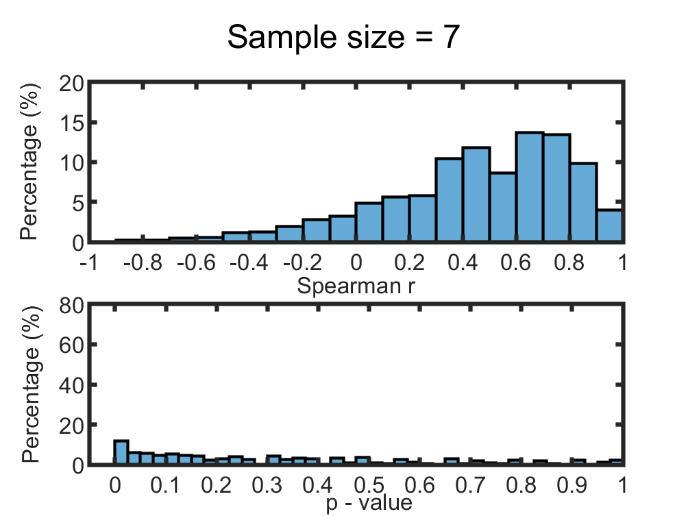

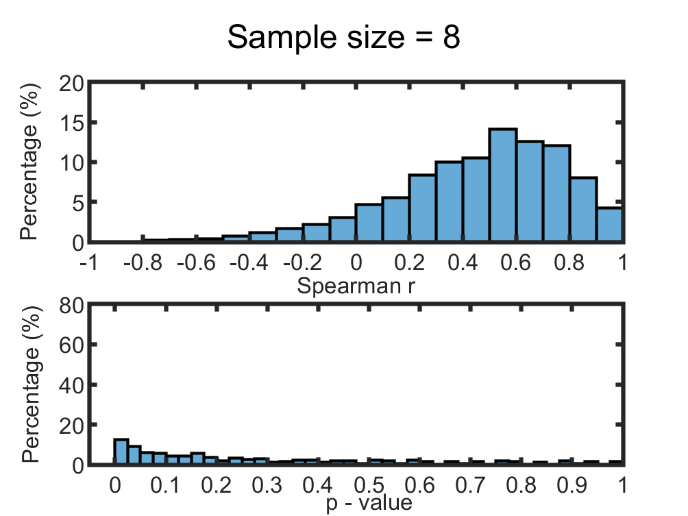


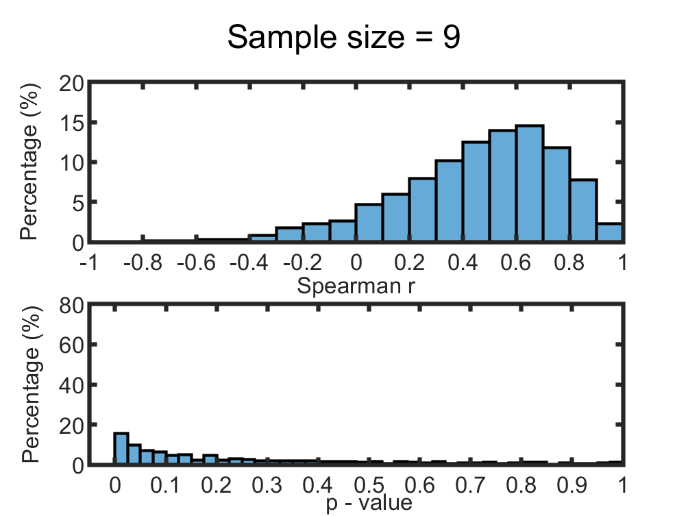

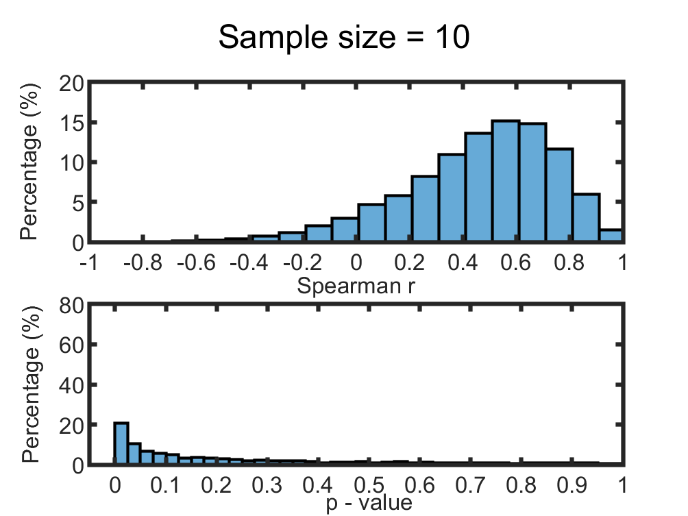


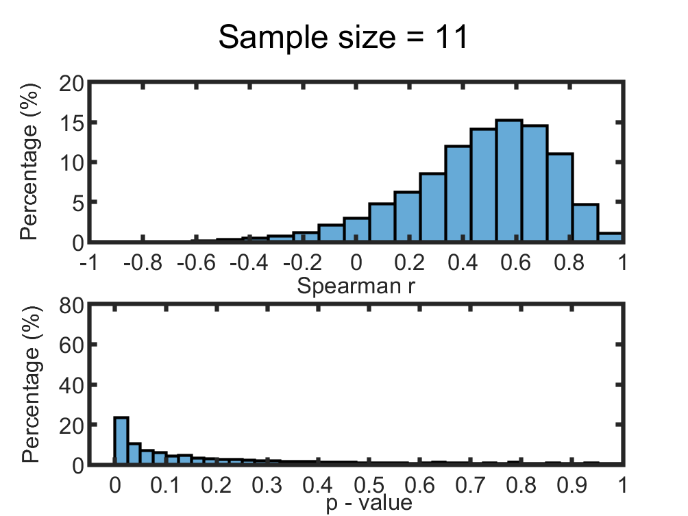

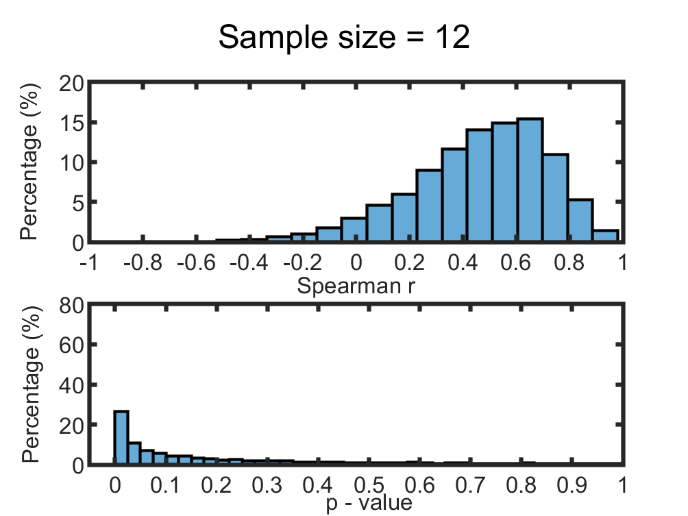


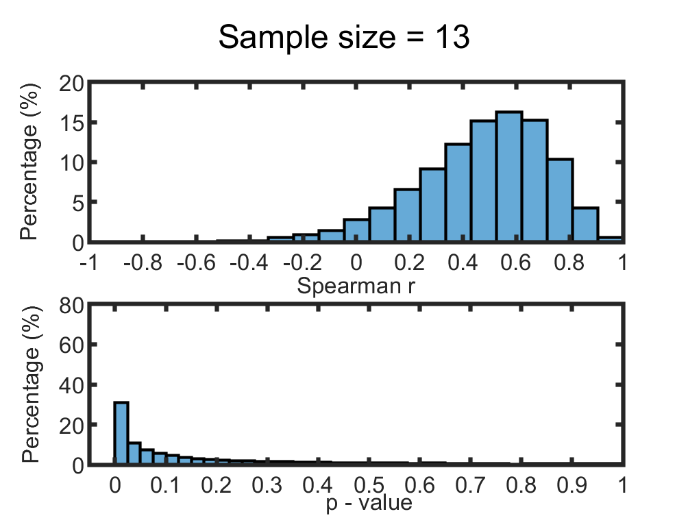

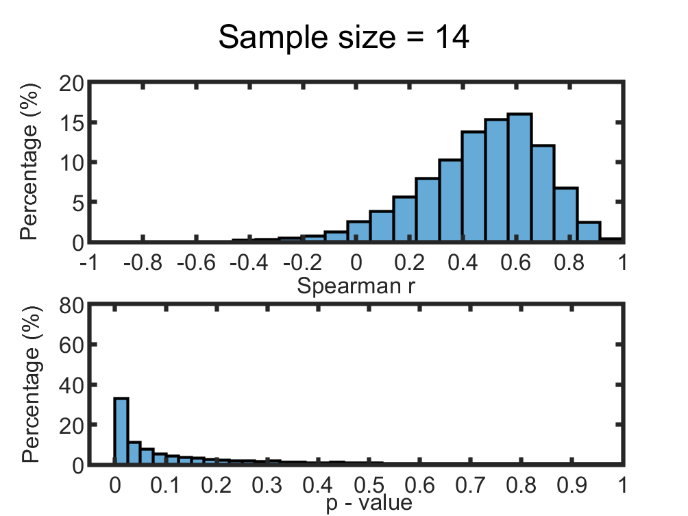


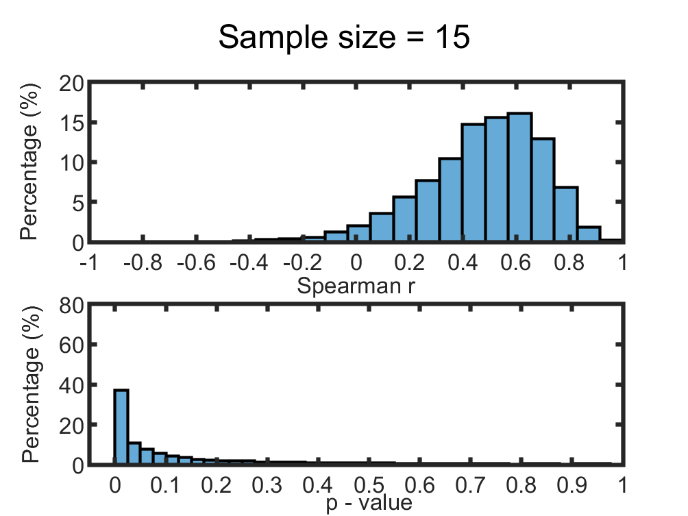

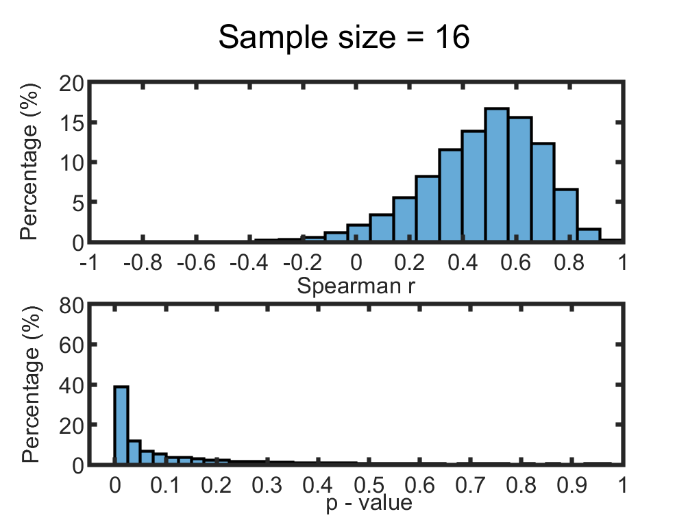


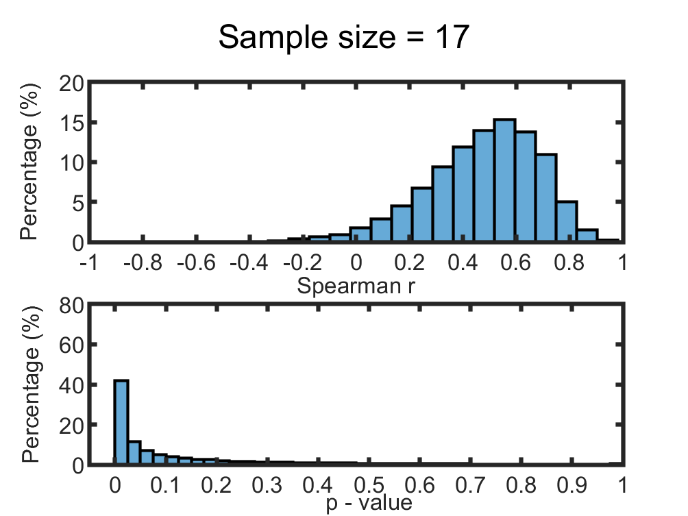

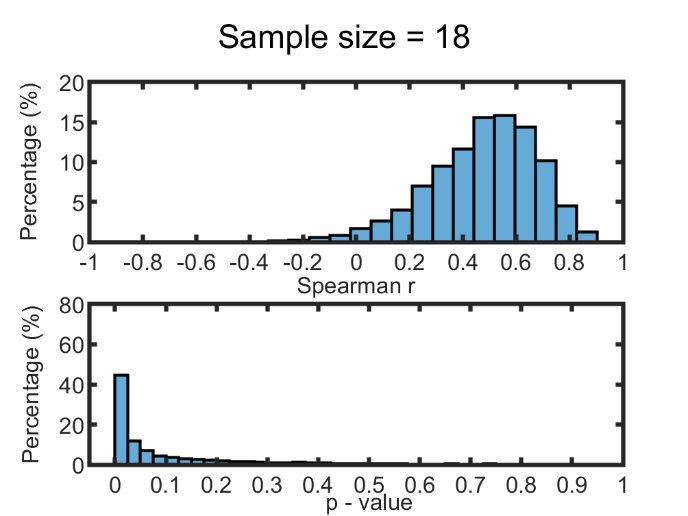


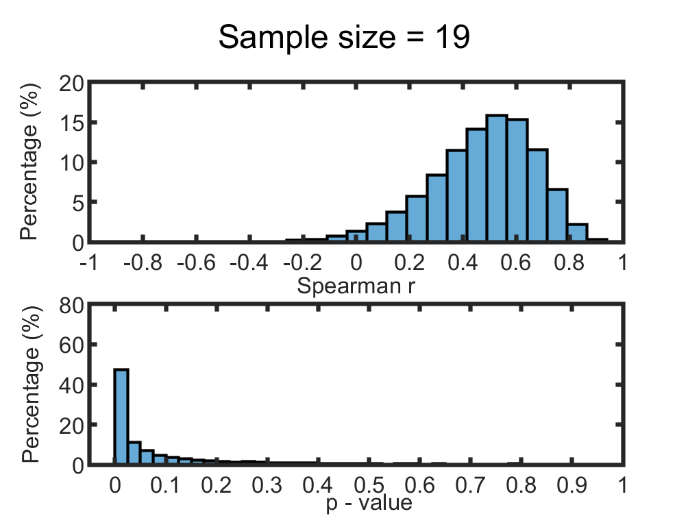

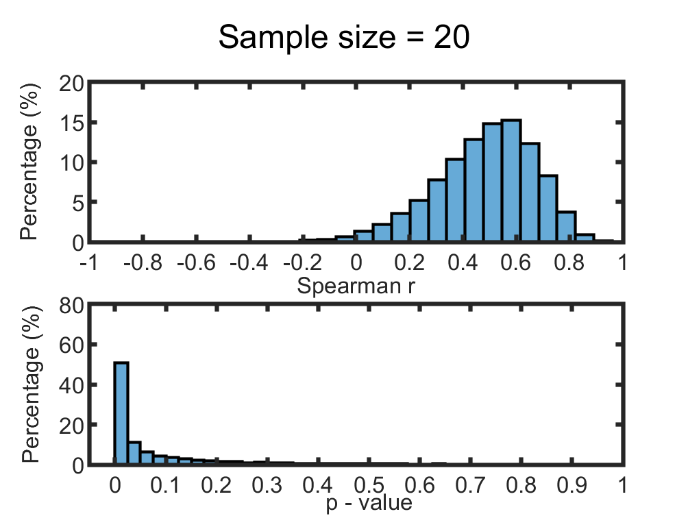


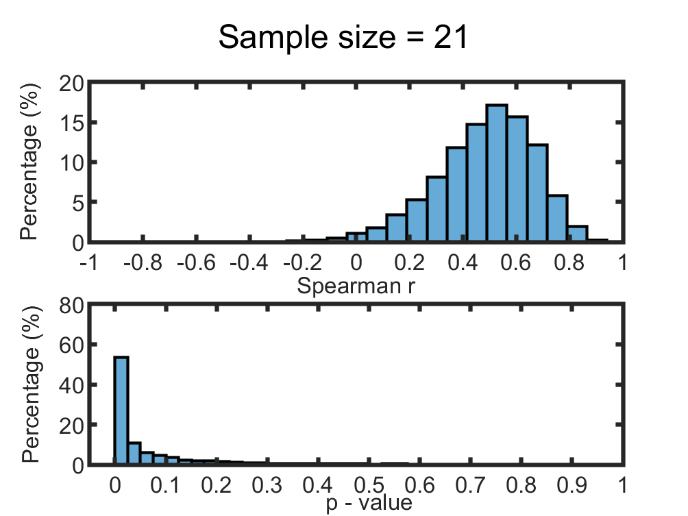

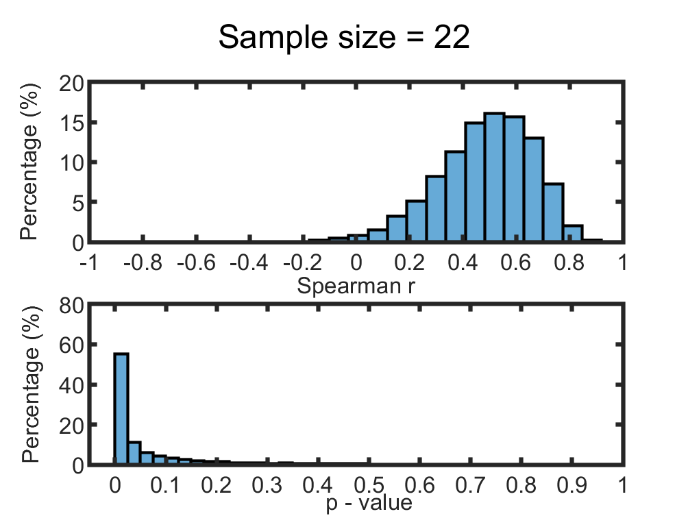


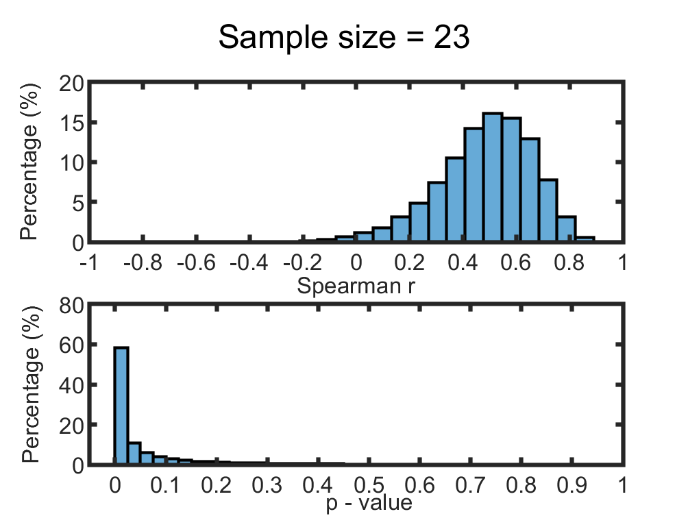

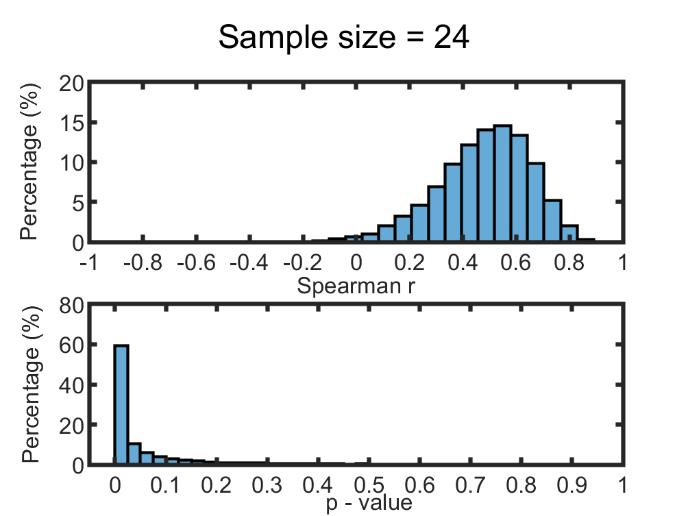


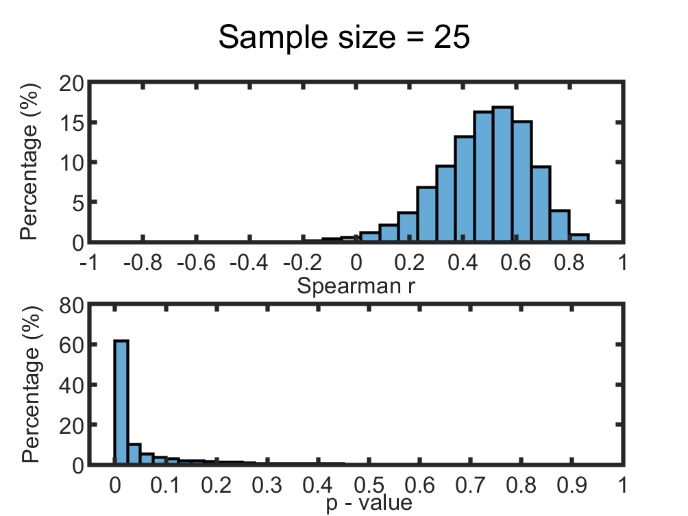

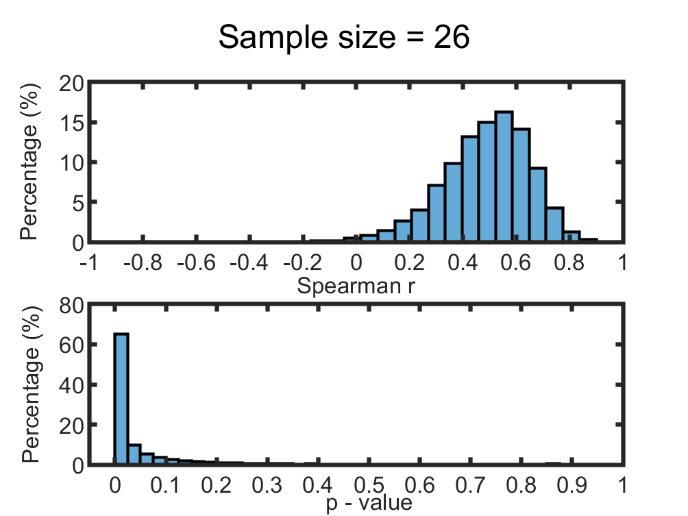


**Supplementary Material 7.** Scatter plots for naïve and expert participant data in the MISC and MIPS tasks using significant accrual rate only in MIPS (N = 26; Figure A) or using all the accrual rates (N = 28; Figure B). The naïve participants were new to psychophysics. The experts were experienced psychophysical observers, including two of the authors, eight staff and PhD students in vision science.

**A B**


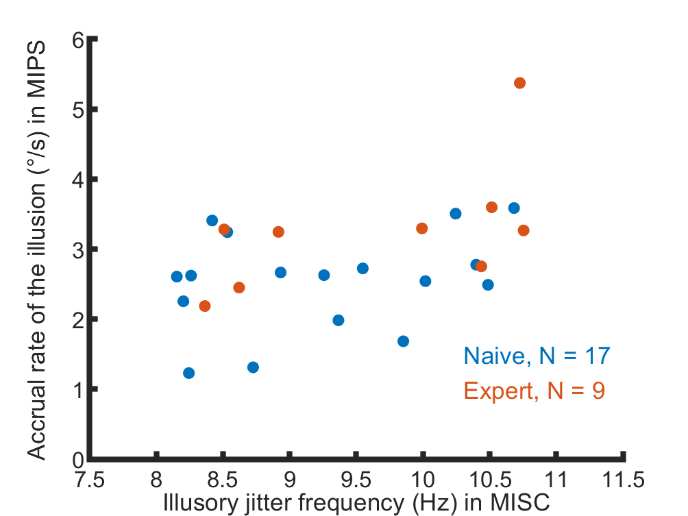

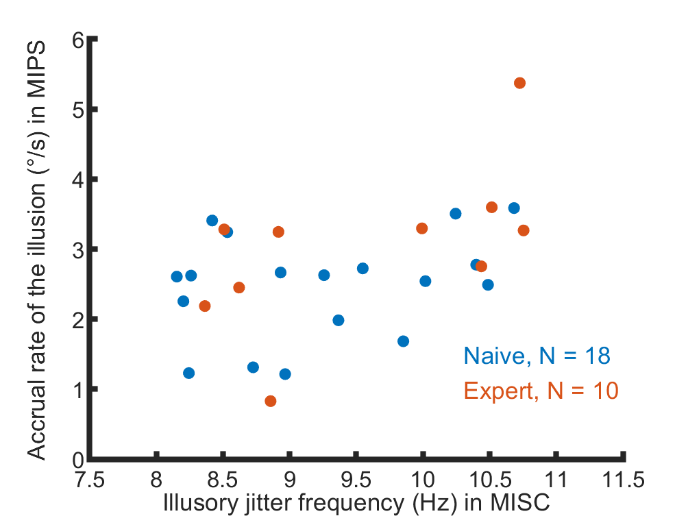

Supplement: Supplementary file 3 [file Data_Sheet_1.docx]
